# Safety and Immunogenicity of an AS03_B_-Adjuvanted Inactivated Tetravalent Dengue Virus Vaccine Administered on Varying Schedules to Healthy U.S. Adults: A Phase 1/2 Randomized Study

**DOI:** 10.4269/ajtmh.19-0738

**Published:** 2020-04-27

**Authors:** Leyi Lin, Kirsten E. Lyke, Michael Koren, Richard G. Jarman, Kenneth H. Eckels, Edith Lepine, Monica A. McArthur, Jeffrey R. Currier, Heather Friberg, Philippe Moris, Paul B. Keiser, Rafael De La Barrera, David W. Vaughn, Robert M. Paris, Stephen J. Thomas, Alexander C. Schmidt

**Affiliations:** 1 Walter Reed Army Institute of Research, Silver Spring, Maryland;; 2 Center for Vaccine Development and Global Health (CVD), University of Maryland, Baltimore, Maryland;; 3 GSK, Rockville, Maryland;; 4 GSK, Rixensart, Belgium

## Abstract

Dengue disease and its causative agents, the dengue viruses (DENV-1–4), cause high morbidity in tropical and subtropical regions. We evaluated three dosing regimens of the investigational tetravalent AS03_B_-adjuvanted dengue-purified inactivated vaccine (DPIV+AS03_B_). In this phase 1/2, observer-blind, placebo-controlled study (NCT02421367), 140 healthy adults were randomized 1:1:2 to receive DPIV+AS03_B_ according to the following regimens: 0–1 month (M), 0–1–6 M, or 0–3 M. Participants received DPIV+AS03_B_ or placebo at M0, M1, M3, and M6 according to their dosing schedule. Primary objectives were 1) to evaluate the safety of DPIV+AS03_B_ for 28 days (D) after each dose; 2) to demonstrate the added value of a booster dose (0–1–6 M versus 0–1 M) based on neutralizing antibody titers to each DENV type (DENV-1–4) at 28 D after the last dose; and, if this objective was met, 3) to demonstrate the benefit of a longer interval between the first and second doses (0–1 M versus 0–3 M). Adverse events (AEs) within 7 D after vaccination tended to be more frequent after DPIV+AS03_B_ doses than placebo; the number of grade 3 AEs was low (≤ 4.5% after DPIV+AS03_B_; ≤ 2.9% after placebo), with no obvious differences across groups. Within 28 D following each dose, the frequency of unsolicited AEs after DPIV+AS03_B_ appeared higher for three-dose (0–1–6 M) than two-dose (0–1 M and 0–3 M) regimens. No serious AEs were considered related to vaccination, and no potential immune-mediated diseases were reported during the study. All three schedules were well tolerated. Both primary immunogenicity objectives were demonstrated. The 0–3 M and 0–1–6 M regimens were more immunogenic than the 0–1 M regimen.

## INTRODUCTION

Dengue, a mosquito-borne disease caused by four types of serologically related but antigenically distinct dengue viruses (DENV-1–4), is one of the leading causes of morbidity in tropical and subtropical regions.^1^ The global burden of dengue has grown substantially, with more than half of the world’s population at risk of infection.^2^

The high global infection rate and disease burden, together with the difficulties in vector control and the limited efficacy of the single licensed dengue vaccine, emphasize the urgent need for additional effective vaccines.^3–6^

The proven effectiveness of other inactivated flavivirus vaccines used for more than 50 years, such as those against Japanese and tick-borne encephalitis, encourage further development of inactivated dengue vaccine candidates.^4^ Several other dengue vaccines are at various stages of clinical development.^7^ Only one vaccine, a chimeric tetravalent construct (CYD-TDV, Dengvaxia, Sanofi Pasteur, Paris, France), is currently licensed. Its indication is limited in many areas to individuals aged 9–45 years (some regulatory agencies restricted further to individuals aged 9–16 years)^8^ with prior DENV infection and living in endemic areas because of the risk of potential disease enhancement in dengue-naive individuals.^5,9^ After AS03-adjuvanted influenza vaccines demonstrated effectiveness and favorable safety profiles,^10^ an investigational inactivated tetravalent dengue virus vaccine (DPIV), formulated with different adjuvants including AS03_B_, was evaluated in a two-dose schedule 1 month (M) apart. Although improvements following non-human primate assessments of CYD-TDV and DPIV+AS03_B_ have been noted, the studies also suggest a risk of enhanced viremia and disease many months following vaccination.^11,12^

Two DPIV+AS03_B_ doses administered 1 M apart were well tolerated and induced robust neutralizing antibody (Nab) titers against all four DENV types in dengue-seronegative^13^ and dengue-seropositive adults.^14^ Although antibody titers waned considerably within 6 M after vaccination in seronegative participants,^13^ they remained high in seropositive participants.^14^ The AS03_B_-adjuvanted DPIV formulation was selected for further development, as AS01_E_- and alum-adjuvanted DPIV formulations were less immunogenic.^13,14^

In this study, we investigated the safety and immunogenicity following vaccination with DPIV+AS03_B_, comparing the 0–1 M regimen with the 0–3 M and 0–1–6 M alternative dosing regimens.

## METHODS

### Study design and participants.

**Figure 1.**
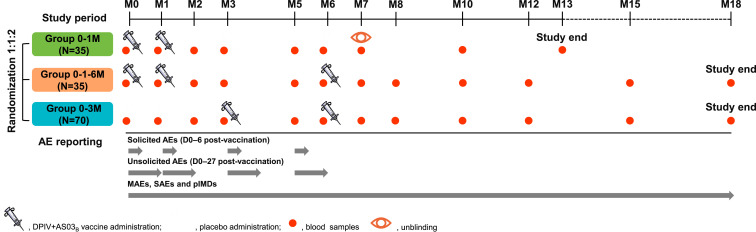
Study design. AEs = adverse events; D = day(s); DPIV+AS03_B_ = adjuvant system 03B-adjuvanted inactivated tetravalent dengue virus vaccine; M = month; MAEs = medically attended AEs; *N* = number of participants in each group included in the total vaccinated cohort; pIMDs = potential immune-mediated diseases; SAEs = serious AEs; 0–1 M = participants receiving two doses of DPIV+AS03_B_ administered 1 M apart, at M0 and M1; 0–3 M = participants receiving two doses of DPIV+AS03_B_ administered 3 M apart, at M3 and M6; 0–1–6 M = participants receiving three doses of DPIV+AS03_B_ with the first two given 1 M apart and the third given 6 M after the first, at M0, M1, and M6. Only immunogenicity blood samples for analyses described here are shown in this figure.

This phase 1/2 randomized, observer-blind study was conducted from August 5, 2015 to June 15, 2017 at two sites in Maryland, United States (Walter Reed Army Institute of Research [WRAIR] and Center for Vaccine Development and Global Health, University of Maryland). Participants were randomized 1:1:2 to receive DPIV+AS03_B_ according to a 0–1 M, 0–1–6 M, or 0–3 M regimen, respectively (Figure 1). Eligible participants were 20- to 49-year-old healthy men and non-pregnant, non-breastfeeding women. Complete inclusion and exclusion criteria are listed in the Supplemental Information.

Informed consent was obtained from each participant at the screening visit, before any study procedures. The study protocol, informed consent, and other relevant documents were reviewed and approved by the WRAIR Institutional Review Board and by the University of Maryland Human Research Protections Office. The study is registered at www.clinicaltrials.gov (NCT02421367). Anonymized individual participant data and study documents can be requested for further research from www.clinicalstudydatarequest.com (study ID 201658).

### Randomization and blinding.

Randomization was performed by GSK using Material Excellence (Matex), a program developed for use in SAS (Cary, NC), with a block size of eight for the day of vaccination (referred to as day [D] 0 hereafter) and a block size of 24 for the rest of the time points. A randomization list of treatment numbers was generated for treatment labeling before material packaging, and a treatment group was associated at random to each treatment number. Blocks were not broken.

Data were collected in an observer-blind manner through the eleventh visit. Study participants and those responsible for the evaluation of any study endpoint were unaware of which experimental vaccination was administered. Administration of the vaccine was performed by unblinded, authorized study personnel at each study site, who did not participate in any of the clinical study evaluation activities.

### Interventions.

Each participant received intramuscular injections into the non-dominant deltoid region whenever possible at M0, M1, M3, and M6 with either DPIV+AS03_B_ or placebo, according to their randomization. Each 0.5-mL DPIV+AS03_B_ dose contained 1 µg of each purified and inactivated virus strain (DENV-1–4) and GSK’s proprietary AS03_B_ adjuvant system containing 5.93 mg α-tocopherol plus squalene in an oil-in-water emulsion. Additional details are provided elsewhere.^13,14^ Each 0.5 mL placebo dose consisted of sodium chloride solution.

### Study objectives.

The primary study objectives were 1) to evaluate the safety and reactogenicity of DPIV+AS03_B_ from D0 to D27 after each dose; 2) to demonstrate the added value of a booster dose (0–1–6 M versus 0–1 M), based on Nab titers to each DENV type at 28 D after the last DPIV+AS03_B_ dose; and, if this objective was met, 3) to demonstrate the benefit of a longer interval between the first and second DPIV+AS03_B_ doses (0–1 M versus 0–3 M).

The secondary objectives were to evaluate the safety of DPIV+AS03_B_ through 12 M after the last dose and to evaluate the Nab titers to each DENV type 28 D after the first DPIV+AS03_B_ dose (groups 0–1 M and 0–1–6 M), 28 and 56 D after the second and third DPIV+AS03_B_ doses (group 0–1–6 M only), as well as 4, 6, 9, and 12 M after the last DPIV+AS03_B_ dose.

Exploratory objectives included the assessment of DENV-specific antibody avidity maturation and of cell-mediated immune (CMI) responses at D0, D28, D35, D84, D168, and D175 (all groups), D224, D504 (groups 0–1–6 M and 0–3 M), and D364 (group 0–1 M) and were performed in a subset of volunteers.

### Safety and reactogenicity assessment.

Adverse events (AEs) were recorded by study participants on diary cards. After assessment of the AEs for seriousness (based on the International Conference on Harmonisation criteria),^15^ relationship to investigational product, severity, and other possible etiologies, the investigator recorded these in the electronic case report forms.

Solicited and unsolicited AEs were collected from D0 through D7 and D27 after each vaccination, respectively. Intensity of AEs was graded on a scale from one (mild) to three (severe). Further details for grading are provided in Supplemental Table S1. Laboratory abnormalities judged as clinically significant by the investigator were recorded as an AE/serious adverse event (SAE) (when meeting the corresponding definition) and graded according to the U.S. Food and Drug Administration guidance.^16^

Serious adverse events, potential immune-mediated diseases (pIMDs), and medically attended AEs were recorded throughout the study. The list of conditions to be recorded as pIMDs is provided in Supplemental Table S2.

### Immunogenicity assessment.

All assays used for the assessment of immunogenicity were conducted at WRAIR, except memory B-cell enzyme-linked immunospot (ELISpot) assays, which were performed at Global Vaccines Clinical Laboratories (Rixensart; Wavre, Belgium). Neutralizing antibody titers specific to each DENV type were measured (17 blood draws for groups 0–1–6 M and 0–3 M, and 14 for the 0–1 M group) by a quantitative microneutralization assay to determine the titer giving 50% reduction in viral infection (MN50). Seropositivity was defined as a Nab titer ≥ 10. Antibody avidity was determined from sera according to a previously described method.^13^

Enzyme-linked immunospot assays and flow cytometry–based intracellular cytokine staining (ICS) for the detection of, respectively, B cells and T cells were performed on cryopreserved peripheral blood mononuclear cells from pre- and post-vaccination time points. Quantification of antigen-specific memory B cells was performed using an adapted method based on a previously developed technique.^17^ Briefly, the method for the detection of total memory B cells consists in the incubation of peripheral blood mononuclear cells that have been differentiated into antibody-secreting cells in plates coated with the antigen of interest (inactivated DENV-1–4), negative control, or anti-human Ig. Then, a conventional immuno-enzymatic procedure was applied to detect antibody/antigen spots enumerating memory B cells.^17^ The results are expressed as the frequencies of antigen-specific memory B cells within the total memory B-cell population.

For ICS, after thawing, the cells were stimulated with overlapping peptide pools representing the capsid, pre-membrane, and envelope structural proteins of all four DENV types to assess the DENV-specific expression of six different T-cell cytokines/functional responses, including CD154, CD107a, interferon-gamma, interleukins 2 and 21, and tumor necrosis factor-alpha. The time periods for evaluation of each safety and immunogenicity endpoint are summarized in Figure 1.

### Statistical analyses.

Safety was evaluated in the total vaccinated cohort (TVC), which included all participants who received at least one dose of study vaccine. Immunogenicity was evaluated in the adapted according-to-protocol (ATP) cohort for immunogenicity, which included at each time point participants who received all doses up to the corresponding time point according to the protocol and had available immunogenicity data. Participants who had pre-vaccination DENV Nab levels above the cut-off for any of four DENV types were not included in the ATP immunogenicity analyses.

A sample size of 140 enrolled participants (35 each in the 0–1 M and 0–1–6 M groups and 70 in the 0–3 M group) was targeted assuming a drop-out rate of 10% to ensure 128 evaluable participants for immunogenicity assessments (32 each in the 0–1 M and 0–1–6 M groups and 64 for 0–3 M group).

The power to demonstrate the added value of the booster dose (second primary objective) for DENV types 1–4 was 90% for a 3.95-fold Nab geometric mean titer (GMT) increase between M7 for the 0–1–6 M group and M2 for the 0–1 M group. The power to demonstrate the added value of a longer interval between doses one and 2 (third primary objective) was 80% for a 2.62-fold Nab GMT increase between M7 for the 0–3 M group and M2 for the pooled 0–1/0–1–6 M group.

Geometric mean titers were calculated by taking the antilog of the mean of the log_10_ titer transformations. Antibody titers below the assay cut-off were given an arbitrary value of half the cut-off.

Primary objective (2) was demonstrated for a given DENV type if the lower limit (LL) of the 95% CI for the fold increase of the GMT for the given DENV type for group 0–1–6 M at 28 D after dose 3 (M7) over group 0–1 M at 28 D after dose 2 (M2) was greater than one.

Primary objective (3) was demonstrated for a given DENV type if the LL of the 95% CI for the fold increase of the GMT for the given DENV type for group 0–3 M at M7 over pooled groups 0–1 M and 0–1–6 M at M2 was greater than one.

All statistical analyses were performed using the SAS Drug Development software package (SAS Institute Inc., Cary, NC).

## RESULTS

### Demographics.

**Figure 2.**
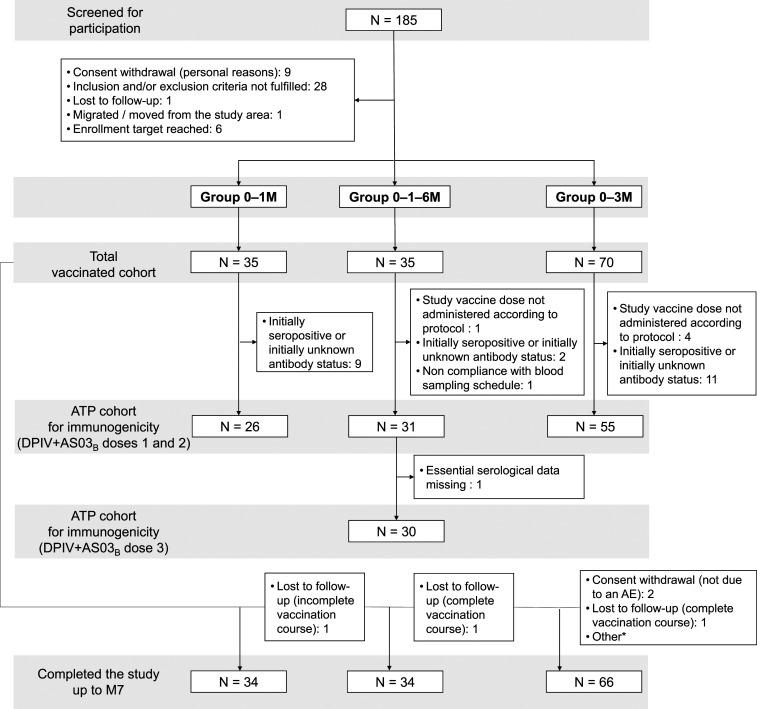
Flow diagram of study participants. AE = adverse event; ATP = according-to-protocol; DPIV+AS03_B_ = adjuvant system 03_B_-adjuvanted inactivated tetravalent dengue virus vaccine; M = month; N = number of participants in each group; 0–1 M = participants receiving two doses of DPIV+AS03_B_ administered 1 M apart, at M0 and M1; 0–3 M = participants receiving two doses of DPIV+AS03_B_ administered 3 M apart, at M3 and M6; 0–1–6 M = participants receiving three doses of DPIV+AS03_B_ with the first two given 1 M apart and the third given 6 M after the first, at M0, M1, and M6. *Non-compliance by participant and outside of window for visit (participation discontinued).

Of the 185 persons screened, 140 were enrolled, randomized, and vaccinated (TVC), and 134 completed the study up to study end (Figure 2). None of the withdrawals was due to AEs or SAEs. Twenty-six (18.6%) vaccinated participants were seropositive for at least one DENV type at baseline and were consequently excluded from the ATP cohort for immunogenicity. The mean age, gender, ethnicity, and geographic ancestry distribution were similar across groups (Supplemental Table S3). All participants received their planned number of DPIV+AS03_B_ doses, except for one (2.9%) participant in the 0–1–6 M group and two (2.9%) in the 0–3 M group; three (8.6%) participants in the 0–1 M group and one (2.9%) in the 0–1–6 M group did not receive the planned number of placebo doses.

### Safety and reactogenicity.

Diary cards were completed by 97.1% and 97.0% of participants after vaccine and placebo doses, respectively. Across groups, the frequency of any (solicited and unsolicited) AE reports in the 7 D following vaccination was 57.6–67.2% after DPIV+AS03_B_ doses and 40.0–47.1% after placebo doses. The incidence of grade 3 AEs reported after DPIV+AS03_B_ and placebo was low, at ≤ 4.5% and ≤ 2.9%, respectively, with no major differences across groups. The incidence of grade 3 AEs related to a vaccine dose was low (2.3–3.0%). There were no grade 3–related AEs reported after any placebo dose (Table 1).

**Table 1:** Incidence of adverse events (solicited and unsolicited) reported during the 7-D (D 0–6) post-vaccination period following DPIV+AS03_B_ and placebo doses (TVC)

			Any grade		Any grade related*		Grade 3		Grade 3 related*	
	Group	*N*	*n*	% (95% CI)	*n*	% (95% CI)	*n*	% (95% CI)	*n*	% (95% CI)
DPIV+AS03_B_ dose 1	0–1 M	35	26	74.3 (56.7–87.5)	26	74.3 (56.7–87.5)	0	0.0 (0.0–10.0)	0	0.0 (0.0–10.0)
	0–1–6 M	35	25	71.4 (53.7–85.4)	25	71.4 (53.7–85.4)	0	0.0 (0.0–10.0)	0	0.0 (0.0–10.0)
	0–1 M/0–1–6 M	70	51	72.9 (60.9–82.8)	51	72.9 (60.9–82.8)	0	0.0 (0.0–5.1)	0	0.0 (0.0–5.1)
	0–3 M	67	38	56.7 (44.0–68.8)	33	49.3 (36.8–61.8)	4	6.0 (1.7–14.6)	2	3.0 (0.4–10.4)
DPIV+AS03_B_ dose 2	0–1 M	34	19	55.9 (37.9–72.8)	19	55.9 (37.9–72.8)	3	8.8 (1.9–23.7)	2	5.9 (0.7–19.7)
	0–1–6 M	33	22	66.7 (48.2–82.0)	17	51.5 (33.5–69.2)	1	3.0 (0.1–15.8)	1	3.0 (0.1–15.8)
	0–1 M/0–1–6 M	67	41	61.2 (48.5–72.9)	36	53.7 (41.1–66.0)	4	6.0 (1.7–14.6)	3	4.5 (0.9–12.5)
	0–3 M	65	38	58.5 (45.6–70.6)	30	46.2 (33.7–59.0)	2	3.1 (0.4–10.7)	1	1.5 (0.0–8.3)
DPIV+AS03_B_ dose 3	0–1–6 M	32	19	59.4 (40.6–76.3)	18	56.3 (37.7–73.6)	2	6.3 (0.8–20.8)	2	6.3 (0.8–20.8)
Overall/DPIV+AS03_B_ dose	0–1 M	69	45	65.2 (52.8–76.3)	45	65.2 (52.8–76.3)	3	4.3 (0.9–12.2)	2	2.9 (0.4–10.1)
	0–1–6 M	100	66	66.0 (55.8–75.2)	60	60.0 (49.7–69.7)	3	3.0 (0.6–8.5)	3	3.0 (0.6–8.5)
	0–1 M/0–1–6 M	137	92	67.2 (58.6–74.9)	87	63.5 (54.9–71.6)	4	2.9 (0.8–7.3)	3	2.2 (0.5–6.3)
	0–3 M	132	76	57.6 (48.7–66.1)	63	47.7 (39.0–56.6)	6	4.5 (1.7–9.6)	3	2.3 (0.5–6.5)
Overall/placebo dose	0–1 M	64	26	40.6 (28.5–53.6)	18	28.1 (17.6–40.8)	1	1.6 (0.0–8.4)	0	–
	0–1–6 M	34	16	47.1 (29.8–64.9)	8	23.5 (10.7–41.2)	1	2.9 (0.1–15.3)	0	–
	0–1 M/0–1–6 M	67	30	44.8 (32.6–57.4)	18	26.9 (16.8–39.1)	1	1.5 (0.0–8.0)	0	–
	0–3 M	140	56	40.0 (31.8–48.6)	36	25.7 (18.7–33.8)	3	2.1 (0.4–6.1)	0	–

DPIV+AS03_B_ = adjuvant system 03_B_-adjuvanted inactivated tetravalent dengue virus vaccine; M= month; *N* = number of patients with at least one documented dose/documented doses; *n*/% = number/percentage of patients/doses followed by at least one type of symptom whatever the study vaccine administered; TVC = total vaccinated cohort; 0–1 M = participants receiving two doses of DPIV+AS03_B_ administered 1 M apart, at M0 and M1; 0–3 M = participants receiving two doses of DPIV+AS03_B_ administered 3 M apart, at M3 and M6; 0–1–6 M = participants receiving three doses of DPIV+AS03_B_ with the first two given 1 M apart and the third given 6 M after the first, at M0, M1, and M6; 0–1 M/0–1–6 M = pooled group.

* Causal relationship to vaccination was assessed by the investigator.

**Figure 3.**
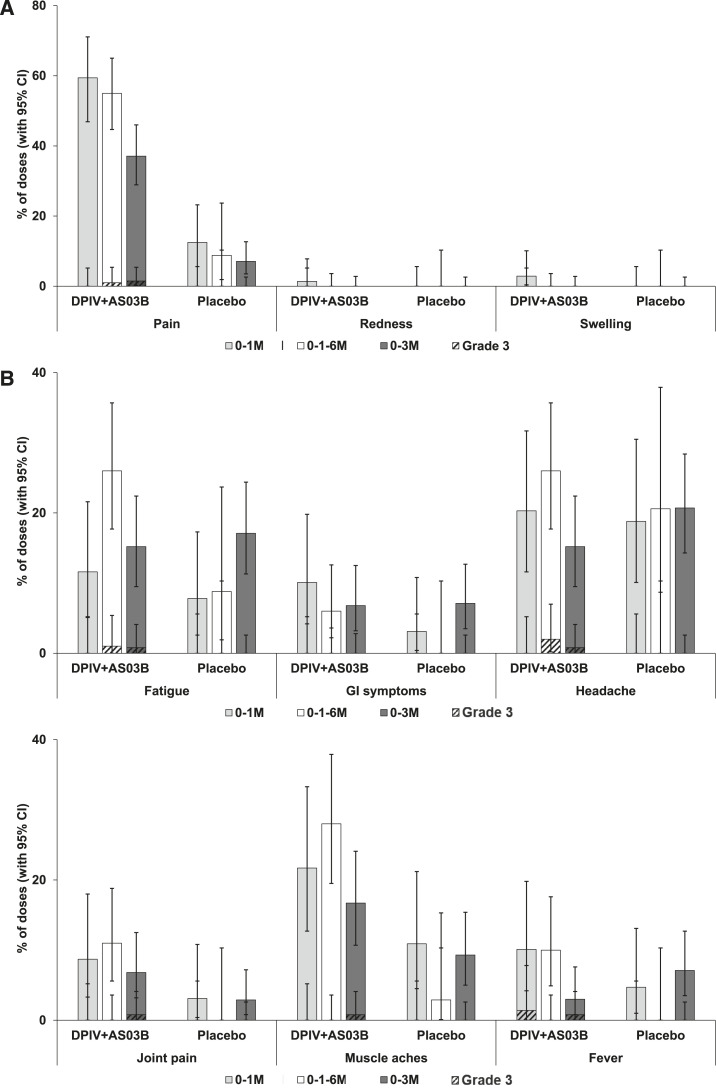
Overall per dose incidence of solicited injection-site (**A**) and general (**B**) adverse events during the 7-D post-vaccination period (TVC). DPIV+AS03_B_ = adjuvant system 03_B_-adjuvanted inactivated tetravalent dengue virus vaccine; GI symtoms = gastrointestinal symptoms (nausea, vomiting, diarrhea, and/or abdominal pain); M = month; TVC = total vaccinated cohort; 0–1 M = participants receiving two doses of DPIV+AS03_B_ administered 1 M apart, at M0 and M1; 0–3 M = participants receiving two doses of DPIV+AS03_B_ administered 3 M apart, at M3 and M6; 0–1–6 M = participants receiving three doses of DPIV+AS03_B_ with the first two given 1 M apart and the third given 6 M after the first, at M0, M1, and M6. Error bars indicate 95% CIs.

Pain was the most common solicited injection site AE within 7 D after DPIV+AS03_B_ vaccination in all groups (reported after 59.4% of doses in the 0–1 M group, 55.0% in the 0–1–6 M group, and 37.1% in the 0–3 M group), as well as after placebo doses (reported after 12.5% of doses in the 0–1 M group, 8.8% in the 0–1–6 M group, and 7.1% in the 0–3 M group). Redness and swelling were reported after ≤ 2.9% of DPIV+AS03_B_ doses and for none of the placebo doses (Figure 3A). Headache, muscle aches, and fatigue were the most common solicited general AEs within 7 D after DPIV+AS03_B_ vaccination as well as after placebo in all groups (Figure 3B). No increases of either injection site or general solicited AEs were observed with successive vaccine doses (Supplemental Table S4). In participants who were seropositive at baseline, the frequency of solicited AEs did not indicate an increased reactogenicity (Supplemental Figure S1).

Within 28 D following doses, the frequency of unsolicited AEs was comparable in the three study groups (three different vaccination schedules) after either DPIV+AS03_B_ or placebo. The number of participants reporting at least one unsolicited AE tended to be higher in the three-dose 0–1–6 M group than in the two-dose 0–1 M group (62.9% versus 42.9%). The frequency of unsolicited AEs was similar in the pooled 0–1 M/0–1–6 M group (44.3%) and the 0–3 M group (45.6%) (Table 2). The most frequently reported unsolicited AE after DPIV+AS03_B_ was anemia (reported by 25.7% of participants in the 0–1–6 M group, 13.2% in the 0–3 M group, and 2.9% in the 0–1 M group). Differences in hemoglobin levels between groups were also observed on the day of first DPIV+AS03_B_ dose administration, with grade 1 or 2 decreases in hemoglobin recorded in 23.5% of participants in the 0–3 M group, 17.1% in the 0–1–6 M group, and 8.6% in the 0–1 M group. The most frequently reported unsolicited AE after placebo was anemia in the 0–1 M group (14.7%), upper respiratory tract infection in the 0–3 M group (8.6%), and anemia and leukocytosis in the 0–1–6 M group (both 5.9%). Upper respiratory tract infection after DPIV+AS03_B_ was reported by 8.6% of participants in the 0–1–6 M group and by 2.9% each in the 0–1 M and 0–3 M groups. After DPIV+AS03_B_, increases in aspartate aminotransferase were reported by 5.7–8.6% of participants in each group, all of which had resolved. Grade 3 or higher aspartate aminotransferase increases after DPIV+AS03_B_ were reported in one participant in the 0–3 M group at 2 M before the first dose, one participant in the 0–3 M group on the day of the first dose, and in two participants in the 0–1 M group at 7 D after the first dose. All except one (which decreased to grade one levels) returned to normal levels by the next blood sampling time point. Of the grade three unsolicited AEs reported after DPIV+AS03_B_, one (2.9%) case of chills in the 0–1 M group was considered related to vaccination by the investigator. No grade 3 unsolicited symptoms with relationship to vaccination were reported after placebo doses. No pIMDs or deaths were reported during the study. Up to study end, a total of 9 SAEs were reported by eight participants: one in the 0–1 M group, two in the 0–1–6 M group, and five in the 0–3 M group. Of these, three occurred within 28 D after any vaccination. None of the SAEs were considered related to vaccination (Supplemental Table S5). No SAEs were reported following placebo administration.

**Table 2:** Incidence of unsolicited adverse events within the 28-D (D 0–27) post-vaccination period following DPIV+AS03_B_ and placebo doses (TVC)

	0–1 M (*N* = 35)		0–1–6 M (*N* = 35)		0–1 M/0–1–6 M (*N* = 70)		0–3 M (*N* = 68)	
	*n*	% (95% CI)	*n*	% (95% CI)	*n*	% (95% CI)	*n*	% (95% CI)
DPIV+AS03_B_								
Any	15	42.9 (26.3–60.6)	22	62.9 (44.9–78.5)	31	44.3 (32.4–56.7)	31	45.6 (33.5–58.1)
Grade 3	1	2.9 (0.1–14.9)	0	0.0 (0.0–10.0)	1	1.4 (0.0–7.7)	0	0.0 (0.0–5.3)
Related*	3	8.6 (1.8–23.1)	2	5.7 (0.7–19.2)	5	7.1 (2.4–15.9)	3	4.4 (0.9–12.4)
Placebo								
Any	12	35.3 (19.7–53.5)	10	29.4 (15.1–47.5)	18	26.5 (16.5–38.6)	33	47.1 (35.1–59.4)

DPIV+AS03_B_ = adjuvant system 03_B_-adjuvanted inactivated tetravalent dengue virus vaccine; M = month; *N* = number of participants with at least one administered dose; *n*/%, number/percentage of participants reporting a symptom at least once; TVC = total vaccinated cohort; 0–1 M = participants receiving two doses of DPIV+AS03_B_ administered 1 M apart, at M0 and M1; 0–3 M = participants receiving two doses of DPIV+AS03_B_ administered 3 M apart, at M3 and M6; 0–1–6 M = participants receiving three doses of DPIV+AS03_B_ with the first two given 1 M apart and the third given 6 M after the first, at M0, M1, and M6; 0–1 M/0–1–6 M = pooled group (0–1 M)/(0–1–6 M).

* Causal relationship to vaccination was assessed by the investigator.

### Immunogenicity.

**Figure 4.**
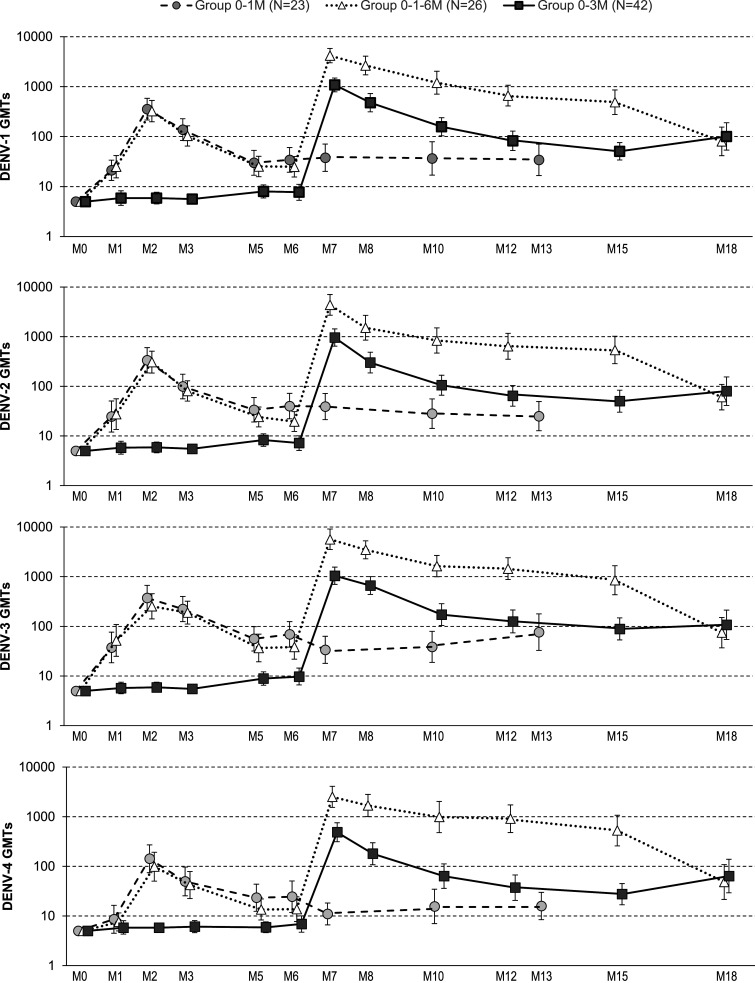
Kinetics of neutralizing antibody responses against DENV-1–4 (ATP cohort for immunogenicity). ATP = according-to-protocol; DENV = dengue virus; GMT = geometric mean titer; N = number of participants in each group; M = month; 0–1 M = participants receiving two doses of adjuvant system 03_B_-adjuvanted inactivated tetravalent dengue virus vaccine (DPIV+AS03_B_) administered one M apart, at M0 and M1; 0–3 M = participants receiving two doses of DPIV+AS03_B_ administered 3 M apart, at M3 and M6; 0–1–6 M = participants receiving three doses of DPIV+AS03_B_ with the first two given 1 M apart and the third given 6 M after the first, at M0, M1, and M6. Error bars depict 95% CIs.

One month after the last vaccine dose, GMTs of Nab to all four DENV types in the 0–1–6 M group were 13.6- to 21.7-fold higher than in the 0–1 M group. One month after the second DPIV+AS03_B_ dose, GMTs were 2.8- to 3.8-fold higher in group 0–3 M than in the pooled group 0–1 M/0–1–6 M (Supplemental Table S6). Importantly, 12 M after the last dose, Nab GMTs in the 0–3 M group were similar or slightly higher than GMTs in the 0–1–6 M group (Figure 4, Supplemental Table S7). For all four DENV types, Nab GMTs peaked at 1 M after the last dose and were highest in the 0–1–6 M group (2,505.4–5,664.6), followed by the 0–3 M (485.8–1,084.4) and 0–1 M (142.1–373.5) groups. Neutralizing antibody GMTs decreased over the 12 M after the last vaccine dose to 48.3–80.3 in the 0–1–6 M group, 63.7–107.4 in the 0–3 M group, and 15.8–76.5 in the 0–1 M group (Figure 4, Supplemental Table S7).

**Figure 5.**
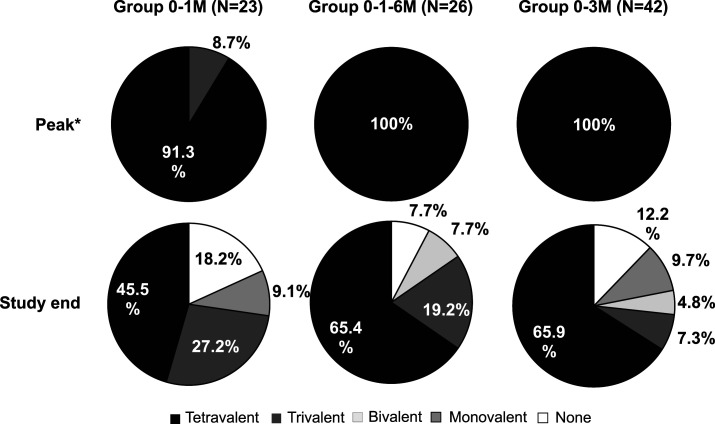
Seroconversion rates to DENV types at peak response and at study end (ATP cohort for immunogenicity). ATP = according-to-protocol; DENV = dengue virus; N = number of participants in each group; 0–1M = participants receiving two doses of adjuvant system 03_B_-adjuvanted inactivated tetravalent dengue virus vaccine (DPIV+AS03_B_) administered 1 M apart, at M0 and M1; 0–3 M = participants receiving two doses of DPIV+AS03_B_ administered 3 M apart, at M3 and M6; 0–1–6 M = participants receiving three doses of DPIV+AS03_B_ with the first two given 1 M apart and the third given 6 M after the first, at M0, M1, and M6. *Peak, 1 M after dose two for groups 0–1 M and 0–3 M and 1 M after dose 3 for group 0–1–6 M.

At study end (12 M after the last DPIV+AS03_B_ dose), tri- or tetravalent responses were more frequent in the 0–1–6 M group (84.6%), than in the 0–3 M (73.2%) and 0–1 M (72.7%) groups. More tetravalent responses were detected in the 0–3 M (65.9%) and 0–1–6 M (65.4%) groups compared to the 0–1 M (45.5%) group (Figure 5). Differences of GMTs at study end were still statistically significant for 0–1–6 M versus 0–1 M for DENV-4 and for 0–3 M versus 0–1 M for DENV-1, DENV-2, and DENV-4 (Supplemental Table S6). In each study group, the decrease in seropositivity rates between the peak of response and study end was greatest for DENV-4 (Supplemental Table S7), suggesting that loss of response to this serotype contributed the most to the loss of tetravalent responses over time.

At pre-vaccination, most participants from the ATP cohort for immunogenicity had no dengue-specific antibody avidity indices for any of the four DENV types, with the exception of DENV-4 in the 0–1 M group, in which the mean avidity index for DENV-4 was 8.18. However, this mean value resulted from one participant in the 0–1 M group with an avidity index of 90.0 and 10 without antibody avidity indices for DENV-4. Through 1 M after the last DPIV+AS03_B_ dose, mean avidity indices increased in all groups and were highest in the 0–1–6 M and 0–3 M groups (Supplemental Table S8).

**Figure 6.**
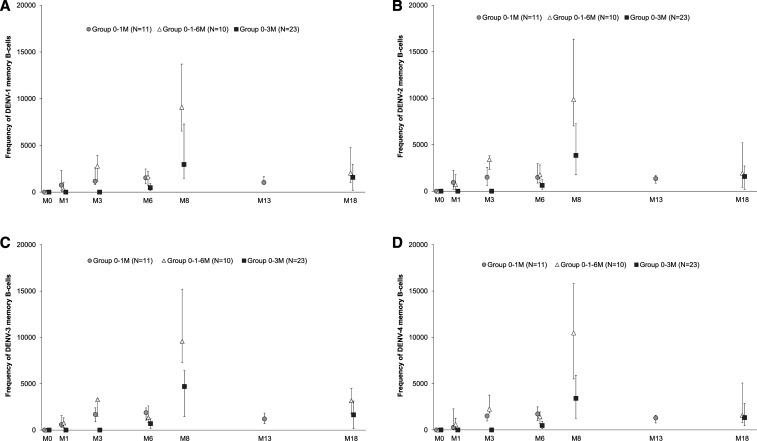
Kinetics for the median (Q1/Q3) frequency (%) of DENV-type–specific memory B cells (by ELISpot) up to study end (ATP cohort for immunogenicity). ATP = according-to-protocol; DENV = dengue virus; ELISpot = enzyme-linked immunospot assay; N = number of participants in each group; M = month; Q1/Q3 = first and third quantiles; 0–1 M = participants receiving two doses of adjuvant system 03_B_-adjuvanted inactivated tetravalent dengue virus vaccine (DPIV+AS03_B_) administered 1 M apart, at M0 and M1; 0–3 M = participants receiving two doses of DPIV+AS03_B_ administered 3 M apart, at M3 and M6; 0–1–6 M = participants receiving three doses of DPIV+AS03_B_ with the first two given 1 M apart and the third given 6 M after the first, at M0, M1, and M6. Error bars depict interquartile ranges.

Pre-vaccination geometric mean frequencies for DENV-specific memory B cells measured by ELISpot assay were similar to assay background (control antigen coating). The DENV-specific memory B-cell frequencies detected at the peak of the response were higher in the 0–1–6 M group than in the other two regimens, which both showed similar levels of responses (Figure 6). Frequencies of DENV-specific memory B cells contracted to a similar magnitude at 12 M after the last DPIV+AS03_B_ dose, irrespective of the schedule. Frequencies of DENV-specific memory B cells were similar for the four DENV types (Figure 6).

In all groups, post-vaccination CD4^+^ T-cell responses were very low overall (Supplemental Figure S2A). Responses were multifunctional, and the predominantly expressed cytokines were interleukin-2 (Supplemental Figure S2B), tumor necrosis factor-alpha (Supplemental Figure S2C), and, to a lesser extent, interferon-gamma. CD4^+^ T-cell responses were slightly higher in the 0–1–6 M group than in the 0–1 M and 0–3 M groups. No CD8^+^ T-cell responses were detected (Supplemental Figure S2D). Although initially DENV-seropositive participants were excluded from the ATP analyses, immunogenicity results in the TVC were similar to those in the ATP cohort for immunogenicity.

## DISCUSSION

All three vaccination regimens were well tolerated and had a clinically acceptable safety profile up to 12 M after the last vaccination, which is consistent with the previous findings with DPIV+AS03_B_ and with the favorable safety profile of AS03-adjuvanted influenza vaccines.^10,13,14^ Although not statistically significant, the number of participants reporting at least one unsolicited AE tended to be higher in the three-dose 0–1–6 M group than in the two-dose 0–1 M group. This difference can be explained by the higher number of doses received by the 0–1–6 M group than the 0–1 M group.

This study met both primary immunogenicity endpoints demonstrating the added value of a third (booster) dose 5 M after the second in a 0–1–6 M regimen (compared with the 0–1 M regimen), and the benefit of a longer interval for a two-dose vaccination regimen (based on Nab titers 28 D after the last dose). With regard to induction of long-lasting Nab titers, as measured at 12 M after the last dose, the 0–1–6 M and 0–3 M regimens appeared equally immunogenic, although the study was not powered to detect such differences. At study end, Nab GMTs in the 0–3 M group were at least 3-fold higher than in the 0–1 M group for three of four DENV types. The titers in the 0–1 M group at 12 M after the last dose were comparable to what was reported previously for a baseline seronegative population.^13^

This study confirmed previous findings that a booster dose increases Nab titers.^13^ Surprisingly, the 0–3 M vaccination regimen was as good as the 0–1–6 M regimen with regard to induction of long-lasting antibody responses. This is important, as a 0–3 M regimen could be more easily implemented than a 0–1–6 M regimen and thus would certainly be preferable. It also shows that two doses of DPIV+AS03_B_ induce a robust and durable immune response. The Nab GMTs observed at 12 M after the last dose in the 0–3 M group are comparable to the GMTs reported for CYD-TDV 1 year after the last dose,^18^ although comparison of Nab titers using different assays should be interpreted cautiously. Importantly, dengue-naive CYD-TDV recipients had a higher rate of severe dengue complications and hospitalizations than dengue-exposed individuals who received the vaccine.^9^ Chimeric tetravalent construct is composed of the live-attenuated yellow fever virus and DENV surface glycoproteins, but lacks nonstructural DENV proteins, with T-cell epitopes that may be essential to induce protective CMI responses against DENV.^19^ Dengue-purified inactivated vaccine is a whole virus, purified, and inactivated vaccine, having potential advantages and disadvantages compared with live-attenuated vaccines with respect to their ability to induce anamnestic or CMI responses, or with respect to persistence of immunogenicity. As CYD-TDV, DPIV does not contain nonstructural DENV proteins. We only detected low, but multifunctional CD4^+^ T-cell responses, and no CD8^+^ T-cell responses. In previous studies, moderately higher CD4^+^ T-cell responses to DPIV were observed, possibly due to the timing of sample collection: 2 M after the last immunization in this study compared with assessing 1 M after immunization previously (H. Friberg, unpublished data). Similar to the inactivated Japanese encephalitis virus vaccine,^20^ a booster dose at 12 M may be needed to induce durable antibody titers. Virus-specific CD4^+^ and CD8^+^ T-cell responses may be an important mechanism for host protection against severe dengue as suggested by studies in mice as well as natural and experimental human infections.^8,21^ By contrast, some prior studies suggested that low-avidity, cross-reactive CD8^+^ T cells are associated with severe dengue.^22^ Current development of the DPIV vaccine candidate is exploring heterologous prime-boost regimens using a tetravalent live-attenuated vaccine together with DPIV, which may induce more durable antibody responses and broaden cellular immune responses, to include CD8^+^ T cells.

This study has limitations, and the data should be interpreted in this context. Although the study was powered to detect statistically significant differences in humoral immune responses between the 0–1 M and 0–1–6 M regimens 28 D after the last dose and between the 0–1 M and 0–3 M regimens 28 D after dose 2, it was not powered to assess differences or non-inferiority in immune responses at 12 M after the last dose. Nonetheless, persistence of immunogenicity appeared similar for the 0–3 M and 0–1–6 M regimens. In the absence of a correlate of protection based on humoral immunity, it is unclear how DENV Nab (by MN50) levels translate to protection against dengue. Immune responses to adjuvanted DPIV in a predominantly dengue-primed population have been previously assessed.^14^ In this study, 18.6% of participants showed serologic evidence of previous flavivirus infection or vaccination at enrollment, including 10 participants with tetravalent dengue responses. Although individuals with self-reported dengue and other flavivirus disease or vaccination were excluded from participation, these results may represent subclinical or undiagnosed dengue, or potentially false positives, as previously observed.^13^ Although these participants were excluded from the per-protocol analysis, immunogenicity was similar when analyzed in all participants who received at least one vaccine dose (data not shown).

In summary, no DPIV+AS03_B_–related safety concerns arose, and the added value of a booster dose 5 M after the second dose of a 0–1 M schedule and the benefit of an increased dose interval (3 M versus 1 M) in a two-dose schedule were demonstrated. Exploratory analyses showed weak but multifunctional CD4^+^ T-cell responses. The predominantly expressed cytokines were interleukin-2, tumor necrosis factor-alpha, and, to a lesser extent, interferon-gamma. Induction of DENV-specific memory B cells was also observed, with a higher response following the three doses in the 0–1–6 M group.

## Supplemental information, tables, and figures

Supplemental materials

## References

[1] World Health Organization, 2018 Dengue vaccine: WHO position paper–September 2018. Wkly Epidemiol Rec 93: 457–476.

[2] Stanaway JD 2016 The global burden of dengue: an analysis from the Global Burden of Disease Study 2013. Lancet Infect Dis 16: 712–723. 26874619 10.1016/S1473-3099(16)00026-8 PMC5012511

[3] Dayan G 2015 Prospective cohort study with active surveillance for fever in four dengue endemic countries in Latin America. Am J Trop Med Hyg 93: 18–23. 26013373 10.4269/ajtmh.13-0663 PMC4497892

[4] Holbrook MR, 2017 Historical perspectives on flavivirus research. Viruses 9: 97. 10.3390/v9050097 PMC5454410 28468299

[5] WHO, 2017 Updated Questions and Answers Related to Information Presented in the Sanofi Pasteur Press Release on 30 November 2017 with Regards to the Dengue Vaccine Dengvaxia®. Available at: http://www.who.int/immunization/diseases/dengue/q_and_a_dengue_vaccine_dengvaxia/en/. Accessed June 7, 2018.

[6] Bowman LR Donegan S McCall PJ, 2016 Is dengue vector control deficient in effectiveness or evidence? Systematic review and meta-analysis. PLoS Negl Trop Dis 10: e0004551. 26986468 10.1371/journal.pntd.0004551 PMC4795802

[7] Torresi J Ebert G Pellegrini M, 2017 Vaccines licensed and in clinical trials for the prevention of dengue. Hum Vaccin Immunother 13: 1059–1072. 28281864 10.1080/21645515.2016.1261770 PMC5443395

[8] Elong Ngono A Shresta S, 2019 Cross-reactive T cell immunity to dengue and zika viruses: new insights into vaccine development. Front Immunol 10: 1316. 31244855 10.3389/fimmu.2019.01316 PMC6579874

[9] Hadinegoro SR 2015 Efficacy and long-term safety of a dengue vaccine in regions of endemic disease. N Engl J Med 373: 1195–1206. 26214039 10.1056/NEJMoa1506223

[10] Cohet C van der Most R Bauchau V Bekkat-Berkani R Doherty TM Schuind A Tavares Da Silva F Rappuoli R Garcon N Innis BL, 2019 Safety of AS03-adjuvanted influenza vaccines: a review of the evidence. Vaccine 37: 3006–3021. 31031030 10.1016/j.vaccine.2019.04.048

[11] Barban V Mantel N De Montfort A Pagnon A Pradezynski F Lang J Boudet F, 2018 Improvement of the dengue virus (DENV) nonhuman primate model via a reverse translational approach based on dengue vaccine clinical efficacy data against DENV-2 and -4. J Virol 92: e00440‒18. 29593041 10.1128/JVI.00440-18 PMC5974474

[12] Borges MB 2019 Detection of post-vaccination enhanced dengue virus infection in macaques: an improved model for early assessment of dengue vaccines. PLoS Pathog 15: e1007721. 31009499 10.1371/journal.ppat.1007721 PMC6497418

[13] Schmidt AC 2017 Phase 1 randomized study of a tetravalent dengue purified inactivated vaccine in healthy adults in the United States. Am J Trop Med Hyg 96: 1325–1337. 28719287 10.4269/ajtmh.16-0634 PMC5462566

[14] Diaz C 2018 Phase I randomized study of a tetravalent dengue purified inactivated vaccine in healthy adults from Puerto Rico. Am J Trop Med Hyg 98: 1435–1443. 29512481 10.4269/ajtmh.17-0627 PMC5953365

[15] International Conference on Harmonisation, 1994 Topic E2A: Clinical Safety Data Management: Definitions and Standards for Expedited Reporting. Available at: http://www.ich.org/fileadmin/Public_Web_Site/ICH_Products/Guidelines/Efficacy/E2A/Step4/E2A_Guideline.pdf. Accessed May 1, 2019.

[16] FDA, 2007 Guidance for Industry. Toxicity Grading Scale for Healthy Adult and Adolescent Volunteers Enrolled in Preventive Vaccine Clinical Trials. Available at: https://www.fda.gov/media/73679/download. Accessed May 1, 2019. 10.1016/j.vaccine.2023.07.072 37532612

[17] Weiskopf D 2015 The human CD8+ T cell responses induced by a live attenuated tetravalent dengue vaccine are directed against highly conserved epitopes. J Virol 89: 120–128. 25320311 10.1128/JVI.02129-14 PMC4301095

[18] Tran NH 2019 Long-term immunogenicity and safety of tetravalent dengue vaccine (CYD-TDV) in healthy populations in Singapore and Vietnam: 4-year follow-up of randomized, controlled, phase II trials. Hum Vaccin Immunother 6: 1–13. 10.1080/21645515.2019.1578595 PMC6816352 30724660

[19] Ahmad Z Poh CL, 2019 The conserved molecular determinants of virulence in dengue virus. Int J Med Sci 16: 355–365. 30911269 10.7150/ijms.29938 PMC6428985

[20] Eder S 2011 Long term immunity following a booster dose of the inactivated Japanese Encephalitis vaccine IXIARO(R), IC51. Vaccine 29: 2607–2612. 21288804 10.1016/j.vaccine.2011.01.058

[21] Grifoni A 2017 Patterns of cellular immunity associated with experimental infection with rDEN2Δ30 (Tonga/74) support its suitability as a human dengue virus challenge strain. J Virol 91: e02133–16. 28148797 10.1128/JVI.02133-16 PMC5375662

[22] Mongkolsapaya J 2003 Original antigenic sin and apoptosis in the pathogenesis of dengue hemorrhagic fever. Nat Med 9: 921–927. 12808447 10.1038/nm887

