# Supplementary material for: Safety and Immunogenicity of an AS03B-Adjuvanted Inactivated Tetravalent Dengue Virus Vaccine Administered on Varying Schedules to Healthy U.S. Adults: A Phase 1/2 Randomized Study
Source: Am J Trop Med Hyg. 2020 Apr 27;103(1):132–41. doi: 10.4269/ajtmh.19-0738 (PMC7356407; doi:10.4269/ajtmh.19-0738)
Supplement: Supplementary file 1 [file tpmd190738.SD1.pdf]

Supplementary Material

A. Inclusion Criteria

Participants were included in the trial if they met all of the following criteria:

- Provided written informed consent.
- Were healthy as established by medical history and clinical examination at study entry.
- The investigator believed they could and would comply with the requirements of the protocol.
- Able to pass Department of Defense base entry requirements, including the possession of a valid government issued identification card (if assigned at the Walter Reed Army Institute of Research).
- Male or non-pregnant, non-breastfeeding female between 20 and 49 years of age (inclusive) at the time of consent.
- Female participants of non-childbearing potential (non-childbearing potential defined as having had one of the following: a tubal ligation at least 3 months prior to enrollment, a hysterectomy, an ovariectomy, or is post-menopausal).
- Female participants of childbearing potential were enrolled in the study, if all of the following applied:
    - Practiced adequate contraception for 30 days prior to vaccination;
    - Had a negative urine pregnancy test on the day of vaccination;
    - Agreed to continue adequate contraception until 3 months after completion of the vaccination series.

B. Exclusion Criteria

Participants were not enrolled if they met any of the following criteria:

- Used any investigational or non-registered product (drug or vaccine) other than the study vaccines/placebo during the period starting 30 days preceding the first dose of study vaccine/placebo and/or planned use during the study period.

- Chronic administration (defined as more than 14 days in total) of immunosuppressants or other immune-modifying drugs during the period starting 90 days prior to the first vaccine/placebo dose (for corticosteroids, prednisone ≥5mg/day or equivalent; inhaled, intranasal and topical steroids were allowed).
- Planned administration or administration of a vaccine/product not planned in the study protocol during the period starting 30 days prior to the first dose of vaccine/placebo until 30 days after the last dose of study vaccine/placebo (routine influenza vaccination were allowed as long as it would not have been not administered within 14 days of the vaccine/placebo and would have been reported to the principal investigator).
- History of dengue infection or dengue illness, or history of flavivirus vaccination (e.g., yellow fever, tick-borne-encephalitis virus, Japanese encephalitis, and dengue).
- Planned administration of any flavivirus vaccine for the entire study duration.
- Concurrent participation in another clinical study, at any time during the study period, in which the participant would have been exposed to an investigational or a non-investigational product (pharmaceutical product or device).
- Confirmed or suspected immunosuppressive or immunodeficient condition, based on medical history and physical examination (no laboratory testing required).
- Family history (first degree relatives only) of congenital or hereditary immunodeficiency.
- Autoimmune disease or history of autoimmune disease.
- History of any reaction or hypersensitivity likely to have been exacerbated by any component of the study vaccine/placebo or related to a study procedure.
- Major congenital defects or serious chronic illness.
- History of any neurological disorders or seizures.

- Diagnosis of excessive daytime sleepiness (unintended sleep episodes during the day present almost daily for at least one month) or narcolepsy; or history of narcolepsy in a participant's parent, sibling, or child

- Acute disease and/or fever (≥37.5°C/99.5°F oral body temperature) at the time of enrollment (a participant with a minor illness such as mild diarrhea, mild upper respiratory infection, etc., without fever, could have been enrolled at the discretion of the investigator).

- Acute or chronic, clinically significant pulmonary, cardiovascular, hepatic or renal functional abnormality, as determined by physical examination or laboratory screening tests.

- Administration of immunoglobulins and/or any blood products during the period starting 90 days preceding the first dose of study vaccine/placebo or planned administration during the study period.

- Recent history of chronic alcohol consumption (more than 2 drinks per day and/or drug abuse) based on participant reported history.

- Pregnant or breastfeeding female or female currently planning to become pregnant or planning to discontinue adequate contraception.

- A planned move to a location that would prohibit participating in the trial until study end for the participant

- Any other condition which, in the opinion of the investigator, prevented the participant from participating in the study.

- Seropositive for hepatitis B surface antigen, hepatitis C virus antibodies, or human immunodeficiency virus antibodies.

- Safety laboratory test results at screening deemed clinically significant or more than grade one deviation from normal.

Table S1. Grading of adverse events

Adverse Event	Intensity Grade	Definition

Adverse Event	Intensity Grade	Definition
**Local injection site adverse event**		
Pain at injection site	1	Mild: Any pain neither interfering with nor preventing normal every day activities.
	2	Moderate: Painful when limb is moved and interferes with every day activities.
	3	Severe: Significant pain at rest. Prevents normal every day activities.
Redness at injection site	1	Greatest surface diameter ≥25 to 50 mm
	2	Greatest surface diameter ≥51 to 100 mm
	3	Greatest surface diameter ≥101 mm
Swelling at injection site	1	Greatest surface diameter ≥25 to 50 mm
	2	Greatest surface diameter ≥51 to 100 mm
	3	Greatest surface diameter ≥101 mm
**Solicited general adverse events**		
Fever	1	37.5 °C – 38.4 °C (99.5 °F – 101.1 °F)
	2	38.5 °C – 38.9 °C (101.2 °F – 102.0 °F)
	3	≥39.0 °C (102.1 °F)
Headache	1	Mild: Headache that is easily tolerated
	2	Moderate: Headache that interferes with normal activity
	3	Severe: Headache that prevents normal activity
Fatigue	1	Mild: Fatigue that is easily tolerated
	2	Moderate: Fatigue that interferes with normal activity
	3	Severe: Fatigue that prevents normal activity
Gastrointestinal symptoms (nausea, vomiting, diarrhea and/or abdominal pain)	1	Mild: Gastrointestinal symptoms that are easily tolerated
	2	Moderate: Gastrointestinal symptoms that interfere with normal activity
	3	Severe: Gastrointestinal symptoms that prevent normal activity
Joint pain	1	Mild: Joint pain that is easily tolerated
	2	Moderate: Joint pain that interferes with normal activity
	3	Severe: Joint pain that prevents normal activity
Muscle aches	1	Mild: Muscle aches that is easily tolerated
	2	Moderate: Muscle aches that interferes with normal activity
	3	Severe: Muscle aches that prevents normal activity
**Unsolicited adverse events**		
All	1	An adverse event which is easily tolerated by the participant, causing minimal discomfort and not interfering with everyday activities
	2	An adverse event which is sufficiently discomforting and interferes with normal everyday activities
	3	An adverse event which prevents normal, everyday activities (i.e., prevent attendance at work/school) and would necessitate the administration of corrective therapy

Table S2. List of conditions to be recorded as potential immune-mediated diseases

**Neuroinflammatory disorders**	**Musculoskeletal disorders**	**Skin disorders**
Cranial nerve disorders, including paralyses/paresis (e.g. Bell's palsy), and neuritis (e.g. optic neuritis)<br>Multiple sclerosis (including variants)<br>Transverse myelitis<br>Guillain-Barré syndrome, (including Miller Fisher syndrome and other variants)<br>Other demyelinating diseases (including acute disseminated encephalomyelitis)<br>Myasthenia gravis (including Lambert-Eaton myasthenic syndrome)<br>Non-infectious encephalitis/ encephalomyelitis<br>Neuritis (including peripheral neuropathies)<br>Narcolepsy	Systemic lupus erythematosus<br>Scleroderma (including, CREST syndrome and morphoea)<br>Systemic sclerosis<br>Dermatomyositis<br>Polymyositis<br>Antisynthetase syndrome<br>Rheumatoid arthritis,<br>Juvenile chronic arthritis, (including Still's disease)<br>Polymyalgia rheumatica<br>Reactive arthritis<br>Psoriatic arthropathy<br>Ankylosing spondylitis (including undifferentiated spondyloarthritides)<br>Relapsing polychondritis<br>Mixed connective tissue disorder	Psoriasis<br>Vitiligo<br>Raynaud's phenomenon<br>Erythema nodosum<br>Autoimmune bullous skin diseases (including pemphigus, pemphigoid and dermatitis herpetiformis)<br>Cutaneous lupus erythematosus<br>Alopecia areata<br>Lichen planus<br>Sweet's syndrome
**Liver disorders**	**Gastrointestinal disorders**	**Metabolic diseases**
Autoimmune hepatitis<br>Primary biliary cirrhosis<br>Primary sclerosing cholangitis<br>Autoimmune cholangitis.	Crohn's disease<br>Ulcerative colitis<br>Ulcerative proctitis<br>Celiac disease	Autoimmune thyroiditis (including Hashimoto's thyroiditis)<br>Grave's or Basedow's disease<br>Diabetes mellitus type I<br>Addison's disease
**Vasculitides**	**Others**	
Large vessels vasculitis including: giant cell arteritis such as Takayasu's arteritis and temporal arteritis.<br>Medium sized and/or small vessels vasculitis including: polyarteritis nodosa, Kawasaki's disease, microscopic polyangiitis, Wegener's granulomatosis, Churg–Strauss syndrome (allergic granulomatous angiitis), Buerger's disease (thromboangiitis obliterans), necrotizing vasculitis and anti-neutrophil cytoplasmic antibody positive vasculitis (type unspecified), Henoch-Schonleinpurpura, Behcet's syndrome, leukocytoclasticvasculitis.	Autoimmune hemolytic anemia<br>Autoimmune thrombocytopenia<br>Antiphospholipid syndrome<br>Pernicious anemia<br>Autoimmune glomerulonephritis (including IgA nephropathy, glomerulonephritis rapidly progressive, membranous glomerulonephritis, membranoproliferative glomerulonephritis, and mesangioproliferative glomerulonephritis)<br>Uveitis<br>Autoimmunemyocarditis/cardiomyopathy<br>Sarcoidosis<br>Stevens-Johnson syndrome<br>Sjögren's syndrome<br>Idiopathic pulmonary fibrosis<br>Goodpasture syndrome	

Table S3. Summary of demographic characteristics - primary analysis (TVC)

Characteristics	Parameters or Categories	0–1 M<br>N = 35	0–1–6 M<br>N = 35	0–3 M<br>N = 70
**Age at dose 1 (years)**	**Mean ± SD**	31.5 ± 7.8	31.6 ± 7.5	32.3 ± 8.1
**Sex, n (%)**	**Female**	20 (57.1)	17 (48.6)	31 (44.3)
	**Male**	15 (42.9)	18 (51.4)	39 (55.7)
**Ethnicity, n (%)**	**American Hispanic or Latino**	3 (8.6)	3 (8.6)	2 (2.9)
	**Not American Hispanic or Latino**	32 (91.4)	32 (91.4)	68 (97.1)
**Geographic Ancestry, n (%)**	**African Heritage / African American**	14 (40)	20 (57.1)	37 (52.9)
	**Asian - Central/South Asian Heritage**	2 (5.7)	0 (0)	2 (2.9)
	**Asian - East Asian Heritage**	0 (0)	0 (0)	3 (4.3)
	**Asian - Japanese Heritage**	0 (0)	1 (2.9)	0 (0)
	**Asian - South East Asian Heritage**	0 (0)	0 (0)	2 (2.9)
	**Native Hawaiian or Other Pacific Islander**	0 (0)	1 (2.9)	0 (0)
	**White - Arabic / North African Heritage**	0 (0)	0 (0)	1 (1.4)
	**White - Caucasian / European Heritage**	15 (42.9)	13 (37.1)	22 (31.4)
	**Other**	4 (11.4)	0 (0)	3 (4.3)

TVC, total vaccinated cohort; M, month; 0–1 M, participants receiving 2 doses of adjuvant system 03_B-adjuvanted inactivated tetravalent dengue virus vaccine (DPIV+$AS03_B$) administered one month apart, at M0 and M1; 0–1–6 M, participants receiving 3 doses of DPIV+$AS03_B$ with first 2 doses given one month apart and the third dose 6 months after the first, at M0, M1 and M6; 0–3 M, participants receiving 2 doses of DPIV+$AS03_B$ administered 3 months apart, at M3 and M6; N, total number of participants; n (%), number (percentage) of participants in a given category; SD, standard deviation.

Table S4. Incidence of solicited adverse events reported during the 7-day (days 0-6) post-vaccination period following DPIV+AS03B doses (TVC)

		0–1 M						0–1–6 M									0–3 M					
		Dose 1			Dose 2			Dose 1			Dose 2			Dose 3			Dose 1			Dose 2		
Symptom	Type	N	n	% (95% CI)	N	n	% (95% CI)	N	n	% (95% CI)	N	n	% (95% CI)	N	n	% (95% CI)	N	n	% (95% CI)	N	n	% (95% CI)
Solicited injection site adverse events																						
Pain	All	35	24	68.6 (50.7–83.1)	34	17	50.0 (32.4–67.6)	35	24	68.6 (50.7–83.1)	33	14	42.4 (25.5–60.8)	32	17	53.1 (34.7–70.9)	67	27	40.3 (28.5–53.0)	65	22	33.8 (22.6–46.6)
	Grade 3	35	0	0.0 (0.0–10.0)	34	0	0.0 (0.0–10.3)	35	0	0.0 (0.0–10.0)	33	1	3.0 (0.1–15.8)	32	0	0.0 (0.0–10.9)	67	2	3.0 (0.4–10.4)	65	0	0.0 (0.0–5.5)
Redness	All	35	0	0.0 (0.0–10.0)	34	1	2.9 (0.1–15.3)	35	0	0.0 (0.0–10.0)	33	0	0.0 (0.0–10.6)	32	0	0.0 (0.0–10.9)	67	0	0.0 (0.0–5.4)	65	0	0.0 (0.0–5.5)
	Grade 3	35	0	0.0 (0.0–10.0)	34	0	0.0 (0.0–10.3)	35	0	0.0 (0.0–10.0)	33	0	0.0 (0.0–10.6)	32	0	0.0 (0.0–10.9)	67	0	0.0 (0.0–5.4)	65	0	0.0 (0.0–5.5)
Swelling	All	35	0	0.0 (0.0–10.0)	34	2	5.9 (0.7–19.7)	35	0	0.0 (0.0–10.0)	33	0	0.0 (0.0–10.6)	32	0	0.0 (0.0–10.9)	67	0	0.0 (0.0–5.4)	65	0	0.0 (0.0–5.5)
	Grade 3	35	0	0.0 (0.0–10.0)	34	0	0.0 (0.0–10.3)	35	0	0.0 (0.0–10.0)	33	0	0.0 (0.0–10.6)	32	0	0.0 (0.0–10.9)	67	0	0.0 (0.0–5.4)	65	0	0.0 (0.0–5.5)
Solicited general adverse events																						
Fatigue	All	35	5	14.3 (4.8–30.3)	34	3	8.8 (1.9–23.7)	35	9	25.7 (12.5–43.3)	33	10	30.3 (15.6–48.7)	32	7	21.9 (9.3–40.0)	67	12	17.9 (9.6–29.2)	65	8	12.3 (5.5–22.8)
	Grade 3	35	0	0.0 (0.0–10.0)	34	1	2.9 (0.1–15.3)	35	0	0.0 (0.0–10.0)	33	0	0.0 (0.0–10.6)	32	1	3.1 (0.1–16.2)	67	1	1.5 (0.0–8.0)	65	0	0.0 (0.0–5.5)
	Related	35	3	8.6 (1.8–23.1)	34	2	5.9 (0.7–19.7)	35	6	17.1 (6.6–33.6)	33	6	18.2 (7.0–35.5)	32	7	21.9 (9.3–40.0)	67	9	13.4 (6.3–24.0)	65	8	12.3 (5.5–22.8)
	Grade 3 related	35	0	0.0 (0.0–10.0)	34	0	0.0 (0.0–10.3)	35	0	0.0 (0.0–10.0)	33	0	0.0 (0.0–10.6)	32	1	3.1 (0.1–16.2)	67	1	1.5 (0.0–8.0)	65	0	0.0 (0.0–5.5)
GI symptoms	All	35	5	14.3 (4.8–30.3)	34	2	5.9 (0.7–19.7)	35	1	2.9 (0.1–14.9)	33	3	9.1 (1.9–24.3)	32	2	6.3 (0.8–20.8)	67	3	4.5 (0.9–12.5)	65	6	9.2 (3.5–19.0)
	Grade 3	35	0	0.0 (0.0–10.0)	34	0	0.0 (0.0–10.3)	35	0	0.0 (0.0–10.0)	33	0	0.0 (0.0–10.6)	32	0	0.0 (0.0–10.9)	67	0	0.0 (0.0–5.4)	65	0	0.0 (0.0–5.5)
	Related	35	2	5.7 (0.7–19.2)	34	1	2.9 (0.1–15.3)	35	1	2.9 (0.1–14.9)	33	3	9.1 (1.9–24.3)	32	2	6.3 (0.8–20.8)	67	2	3.0 (0.4–10.4)	65	5	7.7 (2.5–17.0)
	Grade 3 related	35	0	0.0 (0.0–10.0)	34	0	0.0 (0.0–10.3)	35	0	0.0 (0.0–10.0)	33	0	0.0 (0.0–10.6)	32	0	0.0 (0.0–10.9)	67	0	0.0 (0.0–5.4)	65	0	0.0 (0.0–5.5)
Headache	All	35	11	31.4 (16.9–49.3)	34	3	8.8 (1.9–23.7)	35	10	28.6 (14.6–46.3)	33	9	27.3 (13.3–45.5)	32	7	21.9 (9.3–40.0)	67	12	17.9 (9.6–29.2)	65	8	12.3 (5.5–22.8)
	Grade 3	35	0	0.0 (0.0–10.0)	34	1	2.9 (0.1–15.3)	35	0	0.0 (0.0–10.0)	33	0	0.0 (0.0–10.6)	32	2	6.3 (0.8–20.8)	67	2	3.0 (0.4–10.4)	65	0	0.0 (0.0–5.5)
	Related	35	8	22.9 (10.4–40.1)	34	2	5.9 (0.7–19.7)	35	9	25.7 (12.5–43.3)	33	6	18.2 (7.0–35.5)	32	5	15.6 (5.3–32.8)	67	9	13.4 (6.3–24.0)	65	8	12.3 (5.5–22.8)
	Grade 3 related	35	0	0.0 (0.0–10.0)	34	0	0.0 (0.0–10.3)	35	0	0.0 (0.0–10.0)	33	0	0.0 (0.0–10.6)	32	2	6.3 (0.8–20.8)	67	1	1.5 (0.0–8.0)	65	0	0.0 (0.0–5.5)
Joint Pain	All	35	3	8.6 (1.8–23.1)	34	3	8.8 (1.9–23.7)	35	4	11.4 (3.2–26.7)	33	4	12.1 (3.4–28.2)	32	3	9.4 (2.0–25.0)	67	6	9.0 (3.4–18.5)	65	3	4.6 (1.0–12.9)
	Grade 3	35	0	0.0 (0.0–10.0)	34	0	0.0 (0.0–10.3)	35	0	0.0 (0.0–10.0)	33	0	0.0 (0.0–10.6)	32	0	0.0 (0.0–10.9)	67	1	1.5 (0.0–8.0)	65	0	0.0 (0.0–5.5)
	Related	35	2	5.7 (0.7–19.2)	34	2	5.9 (0.7–19.7)	35	3	8.6 (1.8–23.1)	33	2	6.1 (0.7–20.2)	32	3	9.4 (2.0–25.0)	67	5	7.5 (2.5–16.6)	65	3	4.6 (1.0–12.9)
	Grade 3 related	35	0	0.0 (0.0–10.0)	34	0	0.0 (0.0–10.3)	35	0	0.0 (0.0–10.0)	33	0	0.0 (0.0–10.6)	32	0	0.0 (0.0–10.9)	67	1	1.5 (0.0–8.0)	65	0	0.0 (0.0–5.5)
Muscle Aches	All	35	11	31.4 (16.9–49.3)	34	4	11.8 (3.3–27.5)	35	9	25.7 (12.5–43.3)	33	10	30.3 (15.6–48.7)	32	9	28.1 (13.7–46.7)	67	13	19.4 (10.8–30.9)	65	9	13.8 (6.5–24.7)

	**Grade 3**	35	0	0.0 (0.0–10.0)	34	0	0.0 (0.0–10.3)	35	0	0.0 (0.0–10.0)	33	0	0.0 (0.0–10.6)	32	0	0.0 (0.0–10.9)	67	1	1.5 (0.0–8.0)	65	0	0.0 (0.0–5.5)
	**Relater**	35	10	28.6 (14.6–46.3)	34	3	8.8 (1.9–23.7)	35	8	22.9 (10.4–40.1)	33	8	24.2 (11.1–42.3)	32	9	28.1 (13.7–46.7)	67	12	17.9 (9.6–29.2)	65	9	13.8 (6.5–24.7)
	**Grade 3 related**	35	0	0.0 (0.0–10.0)	34	0	0.0 (0.0–10.3)	35	0	0.0 (0.0–10.0)	33	0	0.0 (0.0–10.6)	32	0	0.0 (0.0–10.9)	67	1	1.5 (0.0–8.0)	65	0	0.0 (0.0–5.5)
**Fever**	**All**	35	3	8.6 (1.8–23.1)	34	4	11.8 (3.3–27.5)	35	4	11.4 (3.2–26.7)	33	3	9.1 (1.9–24.3)	32	3	9.4 (2.0–25.0)	67	2	3.0 (0.4–10.4)	65	2	3.1 (0.4–10.7)
	**≥39.0**	35	0	0.0 (0.0–10.0)	34	1	2.9 (0.1–15.3)	35	0	0.0 (0.0–10.0)	33	0	0.0 (0.0–10.6)	32	0	0.0 (0.0–10.9)	67	1	1.5 (0.0–8.0)	65	1	1.5 (0.0–8.3)
	**Related**	35	2	5.7 (0.7–19.2)	34	3	8.8 (1.9–23.7)	35	4	11.4 (3.2–26.7)	33	2	6.1 (0.7–20.2)	32	3	9.4 (2.0–25.0)	67	1	1.5 (0.0–8.0)	65	2	3.1 (0.4–10.7)
	**≥39.0 related**	35	0	0.0 (0.0–10.0)	34	1	2.9 (0.1–15.3)	35	0	0.0 (0.0–10.0)	33	0	0.0 (0.0–10.6)	32	0	0.0 (0.0–10.9)	67	0	0.0 (0.0–5.4)	65	1	1.5 (0.0–8.3)

DPIV+AS03$_B$, adjuvant system 03$_B$-adjuvanted inactivated tetravalent dengue virus vaccine; TVC, total vaccinated cohort; M, month; N, number of patients with at least one documented dose/documented doses; n/%, number/percentage of patients/doses followed by at least one type of symptom; 95% CI, 95% confidence interval; 0–1 M, participants receiving 2 doses of DPIV+AS03$_B$ administered one month apart, at M0 and M1; 0–1–6 M, participants receiving 3 doses of DPIV+AS03$_B$ with first 2 given one month apart and the third given 6 months after the first, at M0, M1 and M6; 0–3 M, participants receiving 2 doses of DPIV+AS03$_B$ administered 3 months apart, at M3 and M6; GI, gastrointestinal

Note: Causal relationship to vaccination was assessed by the investigator.

Table S5. Listing of SAEs reported up to study end (TVC)

Group	Age (Years)	SAE	Primary System Organ Class	MED type	Dose	Day of onset	Duration	Intensity (grade)	Causality	Outcome
0–1 M	42	Facial bones fracture	Injury, poisoning and procedural complications	HO	4	91	3	3	N	Recovered/resolved
0–1–6 M	28	Alcohol abuse	Psychiatric disorders	MD	4	17	46	2	N	Recovered/resolved
	29	Adjustment disorder	Psychiatric disorders	HO	4	149	11	3	N	Recovered/resolved
	30	Bipolar disorder	Psychiatric disorders	HO	1	25	5	2	N	Recovered/resolved
0–3 M	26	Seizure	Nervous system disorders	ER	3	74	61	1	N	Recovered/resolved with sequelae
	49	Diffuse large B cell lymphoma stage III	Neoplasms benign, malignant and unspecified (incl cysts and polyps)	MD	4	249	214	2	N	Recovered/resolved with sequelae
	47	Facial bones fracture	Injury, poisoning and procedural complications	HO	3	34	3	3	N	Recovered/resolved
	34	Acute HIV infection	Infections and infestations	HO	4	23	9	3	N	Recovered/resolved with sequelae
	27	HIV infection	Infections and infestations	MD	4	307	.	1	N	Not recovered/not resolved

TVC, total vaccinated cohort; SAE, serious adverse event; MED, medically attended visit; HO, hospital; MD, doctor or healthcare provider visit; ER, emergency room; N, negative; 0–1 M, participants receiving 2 doses of adjuvant system 03_B-adjuvanted inactivated tetravalent dengue virus vaccine (DPIV+$AS03_B$) administered one month apart, at M0 and M1; 0–1–6 M, participants receiving 3 doses of DPIV+$AS03_B$ with first 2 given one month apart and the third given 6 months after the first, at M0, M1 and M6; 0–3 M, participants receiving 2 doses of DPIV+$AS03_B$ administered 3 months apart, at M3 and M6.

Table S6. GMT ratios post-last DPIV+AS03$_B$ dose in each group (ATP cohort for immunogenicity)

	One month post-last DPIV+AS03$_B$ dose		
	GMT		GMT ratio (95% CI)
Antibody	0–1–6 M<br>N = 30	0–1 M<br>N = 26	0–1–6 M over 0–1 M
DENV-1	4693.3	345.1	13.6 (8.0–23.2)
DENV-2	4,821.8	300.6	16.0 (8.2–31.2)
DENV-3	6,142.2	366.0	16.8 (8.7–32.3)
DENV-4	2,784.0	128.1	21.7 (10.6–44.4)
	**One month post-second DPIV+AS03$_B$ dose**		
	0–3 M<br>N = 55	0–1 M/0–1–6 M<br>N = 56	0–3 M over 0–1 M/0–1–6 M
DENV-1	987.7	337.7	2.9 (1.9–4.5)
DENV-2	816.6	294.5	2.8 (1.7–4.6)
DENV-3	912.0	308.6	3.0 (1.8–5.0)
DENV-4	428.9	113.1	3.8 (2.1–6.8)
	**12 months post-last DPIV+AS03$_B$ dose**		
	0–1–6 M<br>N = 26	0–1 M<br>N = 22	0–1–6 M over 0–1 M
DENV-1	80.3	34.2	2.3 (0.9–6.1)
DENV-2	60.5	25.0	2.4 (1.0–5.8)
DENV-3	74.7	76.5	1.0 (0.3–2.8)
DENV-4	48.3	15.8	3.1 (1.1–8.5)
	0–1–6 M<br>N = 26	0–3 M<br>N = 41	0–1–6 M over 0–3 M
DENV-1	80.3	100.8	0.8 (0.3–2.0)
DENV-2	60.5	79.9	0.8 (0.3–1.9)
DENV-3	74.7	107.4	0.7 (0.3–1.9)
DENV-4	48.3	63.7	0.8 (0.2–2.4)
	0–3 M<br>N = 41	0–1 M<br>N = 22	0–3 M over 0–1 M
DENV-1	100.8	34.2	2.9 (1.1–8.0)
DENV-2	79.9	25.0	3.2 (1.2–8.8)
DENV-3	107.4	76.5	1.4 (0.5–4.2)
DENV-4	63.7	15.8	4.0 (1.3–12.6)

GMT, geometric mean antibody titer; DPIV+AS03B, adjuvant system 03$_B$-adjuvanted inactivated tetravalent dengue virus vaccine; ATP, according to protocol; 95% CI, 95% confidence interval; N, number of patients with post-vaccination results available; DENV, dengue virus; 0–1 M, participants receiving 2 doses of DPIV+AS03B administered one month apart, at M0 and M1; 0–1–6 M, participants receiving 3 doses of DPIV+AS03B with first 2 given one month apart and the third given 6

months after the first, at M0, M1 and M6; 0–3 M, participants receiving 2 doses of DPIV+AS03B administered 3 months apart, at M3 and M6.

Table S7. Seropositivity rates and GMTs for DENV neutralizing antibodies by MN50 up to study end (ATP cohort for immunogenicity)

Antibody	Group	Timing	N	n	Seropositivity % (95% CI)	GMT Value (95% CI)
**DENV-1**	0–1 M	**M0**	23	0	0.0 (0.0–14.8)	5.0 (5.0–5.0)
		**M1**	23	17	73.9 (51.6–89.8)	21.2 (13.3–33.7)
		**M2**	23	23	100.0 (85.2–100.0)	354.6 (214.3–586.7)
		**M3**	23	23	100.0 (85.2–100.0)	138.1 (83.6–228.2)
		**M5**	23	18	78.3 (56.3–92.5)	29.9 (16.8–53.2)
		**M6**	23	21	91.3 (72.0–98.9)	34.3 (19.4–60.5)
		**M7**	23	19	82.6 (61.2–95.0)	37.9 (20.2–71.1)
		**M10**	23	17	73.9 (51.6–89.8)	36.5 (17.0–78.8)
		**M13**	22	16	72.7 (49.8–89.3)	34.2 (16.6–70.4)
	0–1–6 M	**M0**	26	0	0.0 (0.0–13.2)	5.0 (5.0–5.0)
		**M1**	26	18	69.2 (48.2–85.7)	25.0 (14.9–41.9)
		**M2**	26	26	100.0 (86.8–100.0)	323.9 (198.1–529.6)
		**M3**	26	25	96.2 (80.4–99.9)	103.3 (64.7–165.0)
		**M5**	26	21	80.8 (60.6–93.4)	25.3 (15.8–40.3)
		**M6**	26	21	80.8 (60.6–93.4)	24.9 (15.5–39.9)
		**M7**	26	26	100.0 (86.8–100.0)	4,184.3 (3,004.9–5,826.6)
		**M8**	26	26	100.0 (86.8–100.0)	2,656.6 (1,725.5–4,090.2)
		**M10**	26	26	100.0 (86.8–100.0)	1,201.3 (710.6–2,031.1)
		**M12**	26	26	100.0 (86.8–100.0)	665.1 (412.3–1,072.8)
		**M15**	26	26	100.0 (86.8–100.0)	487.7 (276.4–860.6)
		**M18**	26	24	92.3 (74.9–99.1)	80.3 (41.5–155.5)
	0–3 M	**M0**	42	0	0.0 (0.0–8.4)	5.0 (5.0–5.0)
		**M1**	42	1	2.4 (0.1–12.6)	5.9 (4.2–8.2)
		**M2**	42	2	4.8 (0.6–16.2)	5.9 (4.5–7.7)
		**M3**	42	1	2.4 (0.1–12.6)	5.6 (4.5–7.0)
		**M5**	42	11	26.2 (13.9–42.0)	8.0 (5.9–10.8)
		**M6**	42	7	16.7 (7.0–31.4)	7.7 (5.3–11.1)
		**M7**	42	42	100.0 (91.6–100.0)	1,084.4 (788.2–1,491.9)
		**M8**	42	41	97.6 (87.4–99.9)	478.5 (313.5–730.3)
		**M10**	42	40	95.2 (83.8–99.4)	157.8 (103.9–239.6)
		**M12**	42	38	90.5 (77.4–97.3)	82.4 (52.7–128.6)
		**M15**	41	36	87.8 (73.8–95.9)	50.7 (33.9–75.7)
		**M18**	41	34	82.9 (67.9–92.8)	100.8 (53.6–189.4)
**DENV-2**	0–1 M	**M0**	23	0	0.0 (0.0–14.8)	5.0 (5.0–5.0)
		**M1**	23	13	56.5 (34.5–76.8)	24.6 (12.0–50.8)
		**M2**	23	23	100.0 (85.2–100.0)	334.5 (185.9–601.8)
		**M3**	23	22	95.7 (78.1–99.9)	99.3 (56.1–175.6)

Antibody	Group	Timing	N	n	Seropositivity % (95% CI)	GMT Value (95% CI)
		**M5**	23	18	78.3 (56.3–92.5)	33.6 (19.1–59.3)
		**M6**	23	20	87.0 (66.4–97.2)	39.9 (22.1–72.1)
		**M7**	23	19	82.6 (61.2–95.0)	39.1 (21.3–71.8)
		**M10**	23	18	78.3 (56.3–92.5)	28.1 (14.2–55.7)
		**M13**	22	15	68.2 (45.1–86.1)	25.0 (12.7–49.5)
	0–1–6 M	**M0**	26	0	0.0 (0.0–13.2)	5.0 (5.0–5.0)
		**M1**	26	17	65.4 (44.3–82.8)	27.5 (13.5–56.1)
		**M2**	26	25	96.2 (80.4–99.9)	306.4 (184.5–509.1)
		**M3**	26	25	96.2 (80.4–99.9)	80.5 (50.3–128.8)
		**M5**	26	21	80.8 (60.6–93.4)	24.1 (15.2–38.3)
		**M6**	26	19	73.1 (52.2–88.4)	19.5 (12.3–30.9)
		**M7**	26	26	100.0 (86.8–100.0)	4,369.5 (2,706.9–7,053.3)
		**M8**	26	26	100.0 (86.8–100.0)	1,509.6 (850.9–2,678.3)
		**M10**	26	26	100.0 (86.8–100.0)	836.2 (466.6–1,498.7)
		**M12**	26	25	96.2 (80.4–99.9)	639.7 (351.5–1,164.3)
		**M15**	26	25	96.2 (80.4–99.9)	539.1 (284.8–1,020.4)
		**M18**	26	24	92.3 (74.9–99.1)	60.5 (33.5–109.5)
	0–3 M	**M0**	42	0	0.0 (0.0–8.4)	5.0 (5.0–5.0)
		**M1**	42	1	2.4 (0.1–12.6)	5.8 (4.3–7.8)
		**M2**	42	2	4.8 (0.6–16.2)	5.9 (4.6–7.5)
		**M3**	42	1	2.4 (0.1–12.6)	5.5 (4.5–6.6)
		**M5**	42	13	31.0 (17.6–47.1)	8.3 (6.2–11.1)
		**M6**	42	5	11.9 (4.0–25.6)	7.2 (5.1–10.1)
		**M7**	42	42	100.0 (91.6–100.0)	960.3 (642.0–1436.4)
		**M8**	42	40	95.2 (83.8–99.4)	300.6 (186.3–485.1)
		**M10**	42	39	92.9 (80.5–98.5)	105.5 (66.3–167.6)
		**M12**	42	36	85.7 (71.5–94.6)	64.3 (39.9–103.5)
		**M15**	41	32	78.0 (62.4–89.4)	50.3 (30.2–83.6)
		**M18**	41	32	78.0 (62.4–89.4)	79.9 (41.3–154.4)
**DENV-3**	0–1 M	**M0**	23	0	0.0 (0.0–14.8)	5.0 (5.0–5.0)
		**M1**	23	17	73.9 (51.6–89.8)	37.5 (18.4–76.4)
		**M2**	23	23	100.0 (85.2–100.0)	373.5 (209.4–666.1)
		**M3**	23	22	95.7 (78.1–99.9)	222.8 (124.0–400.6)
		**M5**	23	21	91.3 (72.0–98.9)	56.0 (31.9–98.5)
		**M6**	23	22	95.7 (78.1–99.9)	68.9 (38.2–124.5)
		**M7**	23	18	78.3 (56.3–92.5)	33.6 (17.9–63.0)
		**M10**	23	18	78.3 (56.3–92.5)	38.6 (18.8–79.5)
		**M13**	22	18	81.8 (59.7–94.8)	76.5 (32.7–178.6)
	0–1–6 M	**M0**	26	0	0.0 (0.0–13.2)	5.0 (5.0–5.0)
		**M1**	26	18	69.2 (48.2–85.7)	52.0 (24.9–108.5)
		**M2**	26	25	96.2 (80.4–99.9)	253.1 (141.1–454.2)

Antibody	Group	Timing	N	n	Seropositivity % (95% CI)	GMT Value (95% CI)
		**M3**	26	26	100.0 (86.8–100.0)	188.8 (111.1–320.6)
		**M5**	26	19	73.1 (52.2–88.4)	36.6 (19.3–69.5)
		**M6**	26	21	80.8 (60.6–93.4)	38.3 (21.8–67.3)
		**M7**	26	26	100.0 (86.8–100.0)	5,664.6 (3,507.2–9,148.9)
		**M8**	26	26	100.0 (86.8–100.0)	3,487.9 (2,295.9–5,298.7)
		**M10**	26	26	100.0 (86.8–100.0)	1,631.0 (993.4–2,677.6)
		**M12**	26	26	100.0 (86.8–100.0)	1,449.7 (877.1–2,396.1)
		**M15**	26	25	96.2 (80.4–99.9)	850.9 (433.3–1,670.8)
		**M18**	26	22	84.6 (65.1–95.6)	74.7 (36.9–151.1)
	0–3 M	**M0**	42	0	0.0 (0.0–8.4)	5.0 (5.0–5.0)
		**M1**	42	1	2.4 (0.1–12.6)	5.7 (4.4–7.5)
		**M2**	42	2	4.8 (0.6–16.2)	5.9 (4.7–7.5)
		**M3**	42	1	2.4 (0.1–12.6)	5.5 (4.5–6.8)
		**M5**	42	13	31.0 (17.6–47.1)	8.9 (6.5–12.1)
		**M6**	42	13	31.0 (17.6–47.1)	9.7 (6.6–14.4)
		**M7**	42	42	100.0 (91.6–100.0)	1,045.2 (701.6–1557.1)
		**M8**	42	41	97.6 (87.4–99.9)	663.1 (439.7–1,000.1)
		**M10**	42	39	92.9 (80.5–98.5)	172.3 (103.5–286.8)
		**M12**	42	37	88.1 (74.4–96.0)	125.5 (74.0–212.7)
		**M15**	41	37	90.2 (76.9–97.3)	88.6 (53.1–147.6)
		**M18**	41	32	78.0 (62.4–89.4)	107.4 (54.3–212.6)
**DENV-4**	0–1 M	**M0**	23	0	0.0 (0.0–14.8)	5.0 (5.0–5.0)
		**M1**	23	4	17.4 (5.0–38.8)	8.6 (4.5–16.4)
		**M2**	23	21	91.3 (72.0–98.9)	142.1 (74.7–270.6)
		**M3**	23	18	78.3 (56.3–92.5)	49.6 (25.4–96.6)
		**M5**	23	16	69.6 (47.1–86.8)	23.3 (12.4–43.6)
		**M6**	23	15	65.2 (42.7–83.6)	24.4 (11.7–50.7)
		**M7**	23	9	39.1 (19.7–61.5)	11.0 (6.6–18.2)
		**M10**	23	10	43.5 (23.2–65.5)	15.5 (7.0–34.5)
		**M13**	22	11	50.0 (28.2–71.8)	15.8 (8.4–29.9)
	0–1–6 M	**M0**	26	0	0.0 (0.0–13.2)	5.0 (5.0–5.0)
		**M1**	26	5	19.2 (6.6–39.4)	7.6 (5.0–11.5)
		**M2**	26	24	92.3 (74.9–99.1)	98.4 (50.3–192.4)
		**M3**	26	20	76.9 (56.4–91.0)	42.0 (22.4–78.7)
		**M5**	26	14	53.8 (33.4–73.4)	13.5 (8.3–22.0)
		**M6**	26	11	42.3 (23.4–63.1)	13.7 (7.7–24.3)
		**M7**	26	26	100.0 (86.8–100.0)	2,505.4 (1,542.4–4,069.4)
		**M8**	26	26	100.0 (86.8–100.0)	1,680.8 (1,008.0–2,802.7)
		**M10**	26	25	96.2 (80.4–99.9)	982.8 (476.3–2,027.7)
		**M12**	26	25	96.2 (80.4–99.9)	909.9 (478.3–1,730.9)
		**M15**	26	25	96.2 (80.4–99.9)	524.7 (258.4–1,065.7)

Antibody	Group	Timing	N	n	Seropositivity % (95% CI)	GMT Value (95% CI)
		**M18**	26	17	65.4 (44.3–82.8)	48.3 (21.6–108.0)
	0–3 M	**M0**	42	0	0.0 (0.0–8.4)	5.0 (5.0–5.0)
		**M1**	42	1	2.4 (0.1–12.6)	5.8 (4.3–8.0)
		**M2**	42	2	4.8 (0.6–16.2)	5.8 (4.7–7.1)
		**M3**	42	2	4.8 (0.6–16.2)	6.1 (4.6–8.1)
		**M5**	42	2	4.8 (0.6–16.2)	5.9 (4.6–7.6)
		**M6**	42	3	7.1 (1.5–19.5)	6.9 (4.7–10.1)
		**M7**	42	42	100.0 (91.6–100.0)	485.8 (313.0–754.1)
		**M8**	42	40	95.2 (83.8–99.4)	179.5 (107.7–299.2)
		**M10**	42	33	78.6 (63.2–89.7)	63.4 (36.0–111.8)
		**M12**	42	27	64.3 (48.0–78.4)	37.2 (20.6–67.1)
		**M15**	41	26	63.4 (46.9–77.9)	27.5 (16.8–44.9)
		**M18**	41	27	65.9 (49.4–79.9)	63.7 (29.3–138.2)

GMT, geometric mean antibody titer calculated on all participants; ATP, according-to-protocol; M, month; DENV, dengue virus; MN50, microneutralization titer giving 50% reduction in viral infection; N, number of participants with available results; n/%, number/percentage of participants with titer equal to or above endpoint determination equivalent to antibody seropositivity; 95% CI, 95% confidence interval; 0–1 M, participants receiving 2 doses of adjuvant system 03_B-adjuvanted inactivated tetravalent dengue virus vaccine (DPIV+$AS03_B$) administered one month apart, at M0 and M1; 0–1–6 M, participants receiving 3 doses of DPIV+$AS03_B$ with first 2 given one month apart and the third given 6 months after the first, at M0, M1 and M6; 0–3 M, participants receiving 2 doses of DPIV+$AS03_B$ administered 3 months apart, at M3 and M6.

Table S8. Mean avidity index (ATP cohort for immunogenicity)

Group	Timing	N	Mean Avidity Index			
			DENV-1	DENV-2	DENV-3	DENV-4
0–1 M	**M0**	11	0.00	0.00	0.00	8.18
	**M2**	11	42.45	44.09	58.64	61.64
0–1–6 M	**M0**	11	0.00	0.00	0.00	0.00
	**M7**	11	102	102	107.73	113.27
0–3 M	**M2**	25	0.00	3.52	0.00	1.80
	**M7**	25	92.76	91.68	93.84	99.92

ATP, according-to-protocol; DENV, dengue virus; N, number of participants with available results; M, month; 0–1 M, participants receiving 2 doses of adjuvant system 03_B-adjuvanted inactivated tetravalent dengue virus vaccine (DPIV+$AS03_B$) administered one month apart, at M0 and M1; 0–1–6 M, participants receiving 3 doses of DPIV+$AS03_B$ with first 2 given one month apart and the third given 6 months after the first, at M0, M1 and M6; 0–3 M, participants receiving 2 doses of DPIV+$AS03_B$ administered 3 months apart, at M3 and M6.

Figure S1. Overall per dose incidence of solicited injection-site (A) and general (B) adverse events during the 7-day post-vaccination period (initially dengue seropositive TVC participants)

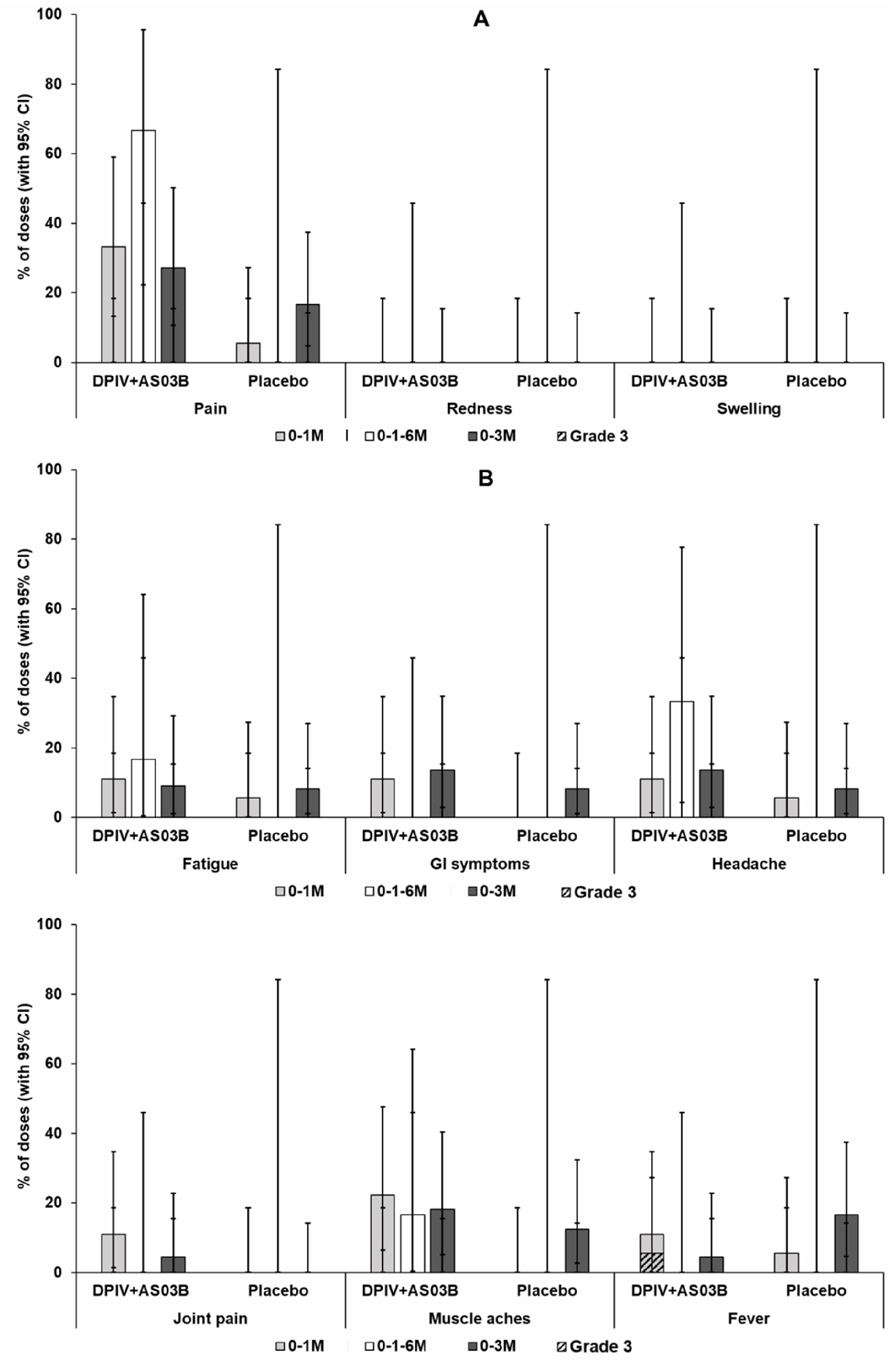

TVC, total vaccinated cohort; DPIV+AS03$_B$, adjuvant system 03$_B$-adjuvanted inactivated tetravalent dengue virus vaccine; M, month; 0–1 M, participants receiving 2 doses of DPIV+AS03$_B$ administered

one month apart, at M0 and M1; 0–1–6 M, participants receiving 3 doses of DPIV+$AS03_B$ with first 2 given one month apart and the third given 6 months after the first, at M0, M1 and M6; 0–3 M, participants receiving 2 doses of DPIV+$AS03_B$ administered 3 months apart, at M3 and M6; error bars indicate 95% confidence intervals (CI); GI symtoms, gastrointestinal symptoms (nausea, vomiting, diarrhea and/or abdominal pain).

Figure S2. DENV-type-specific CD4+ and CD8+ T-cell responsesup to study end (ATP cohort for immunogenicity)

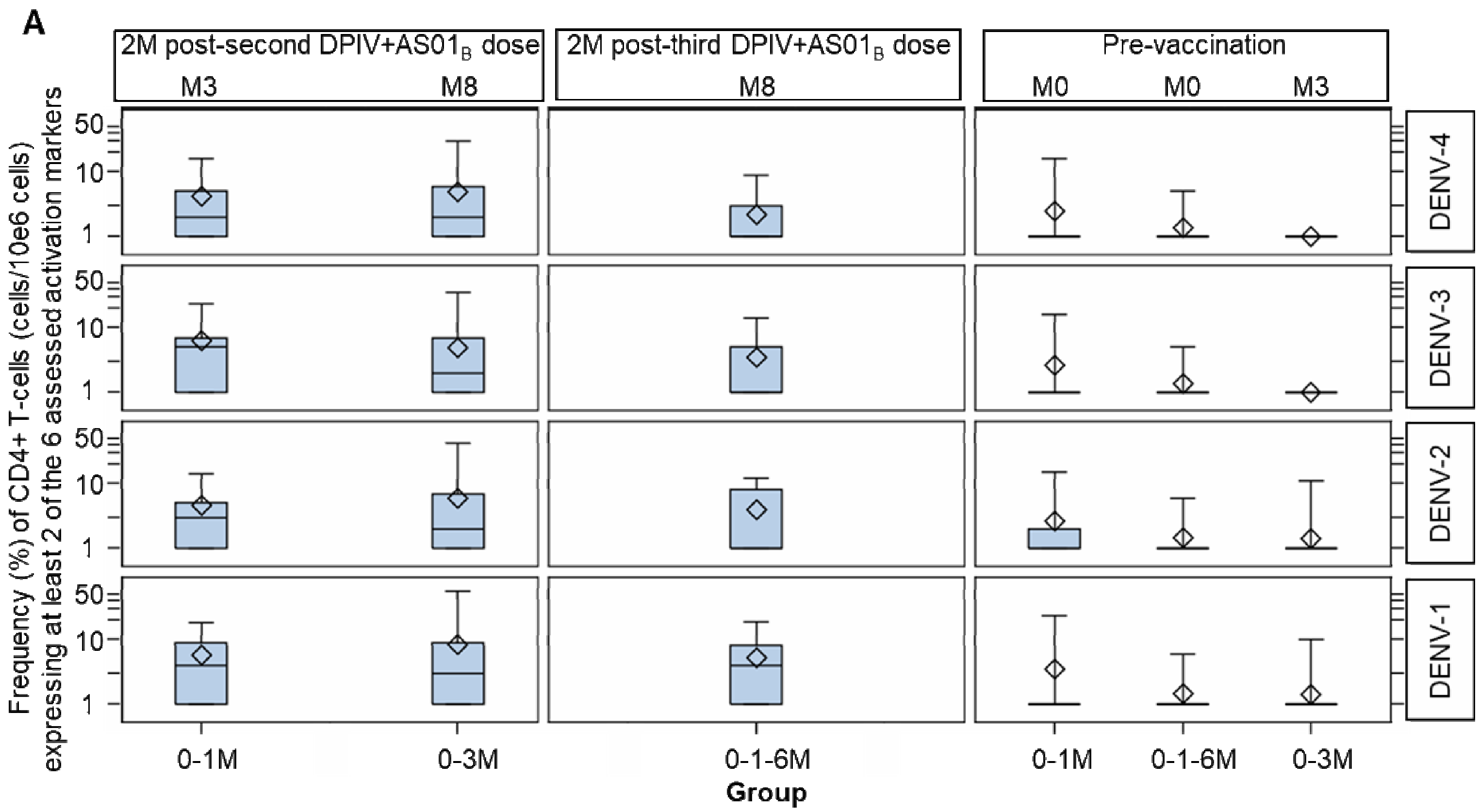

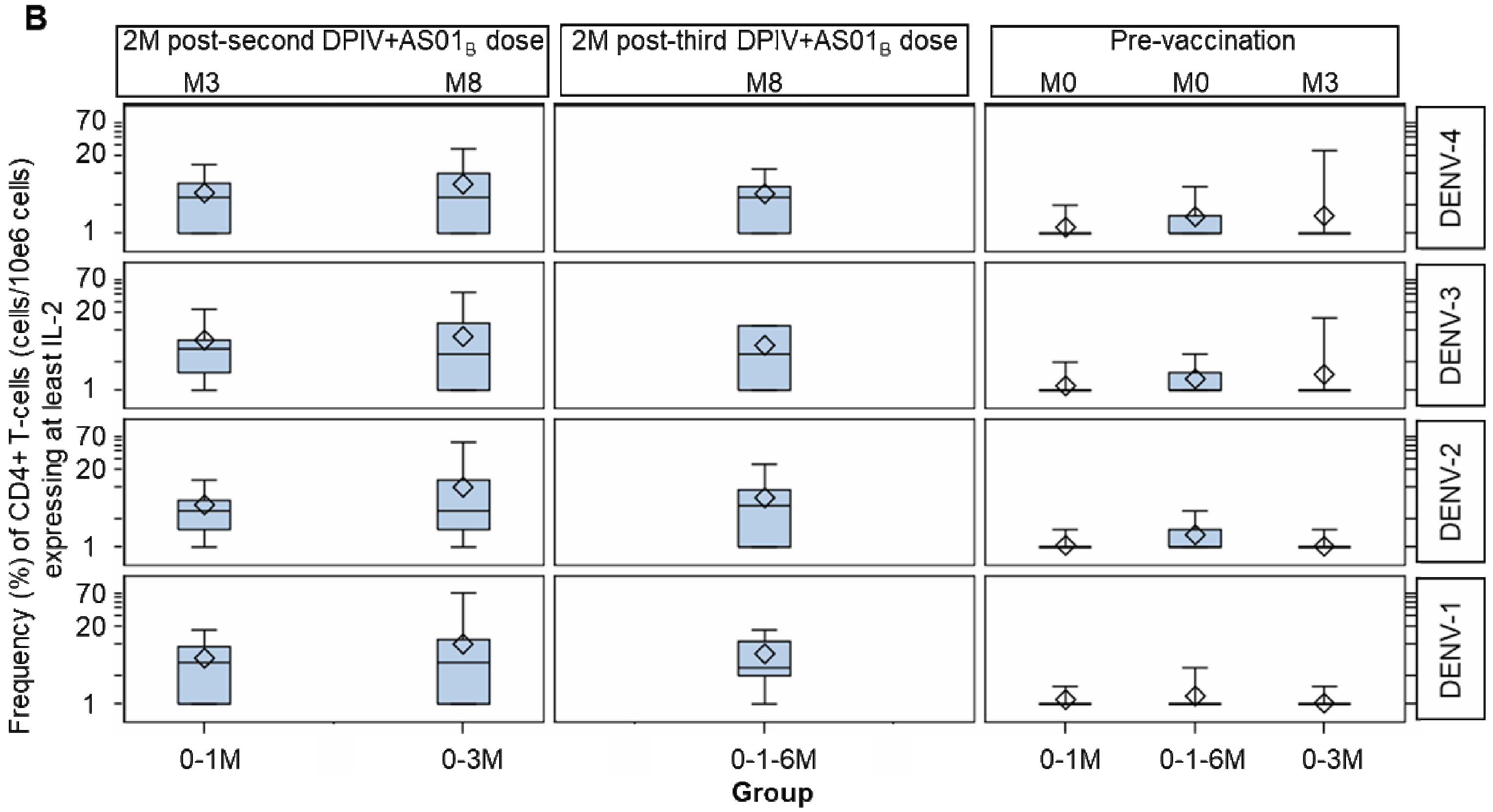
B
2M post-second DPIV+AS01B dose
M3
M8
2M post-third DPIV+AS01B dose
M8
Pre-vaccination
M0
M0
M3
70
20
1
DENV-4
DENV-3
DENV-2
DENV-1
Frequency (%) of CD4+ T-cells (cells/10e6 cells) expressing at least IL-2
0-1M
0-3M
0-1-6M
0-1M
0-1-6M
0-3M
Group

C

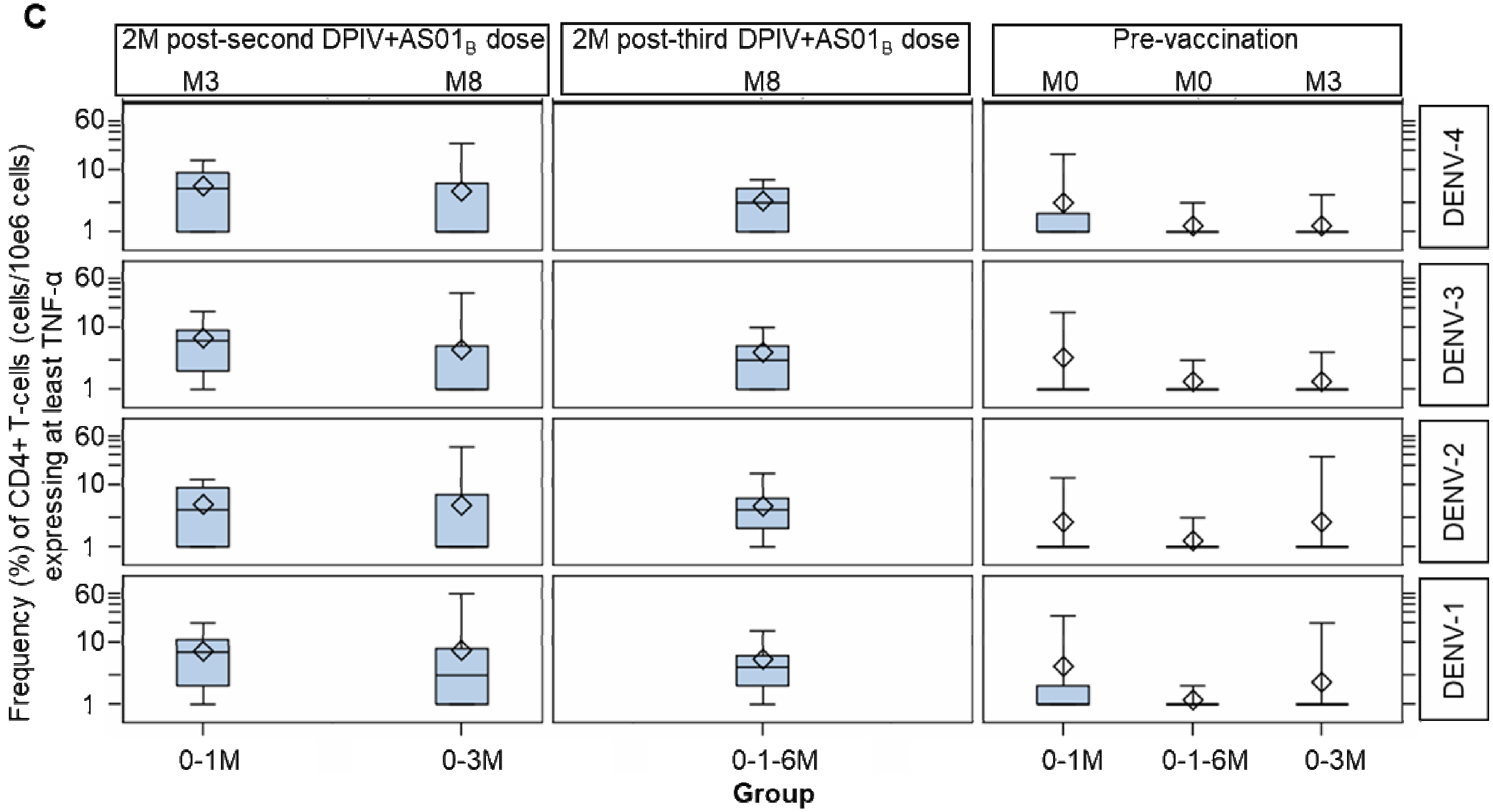

2M post-second DPIV+AS01B dose
M3
M8
2M post-third DPIV+AS01B dose
M8
Pre-vaccination
M0
M0
M3
Frequency (%) of CD4+ T-cells (cells/10e6 cells) expressing at least TNF-α
60
10
1
DENV-4
DENV-3
DENV-2
DENV-1
0-1M
0-3M
0-1-6M
0-1M
0-1-6M
0-3M
Group

D

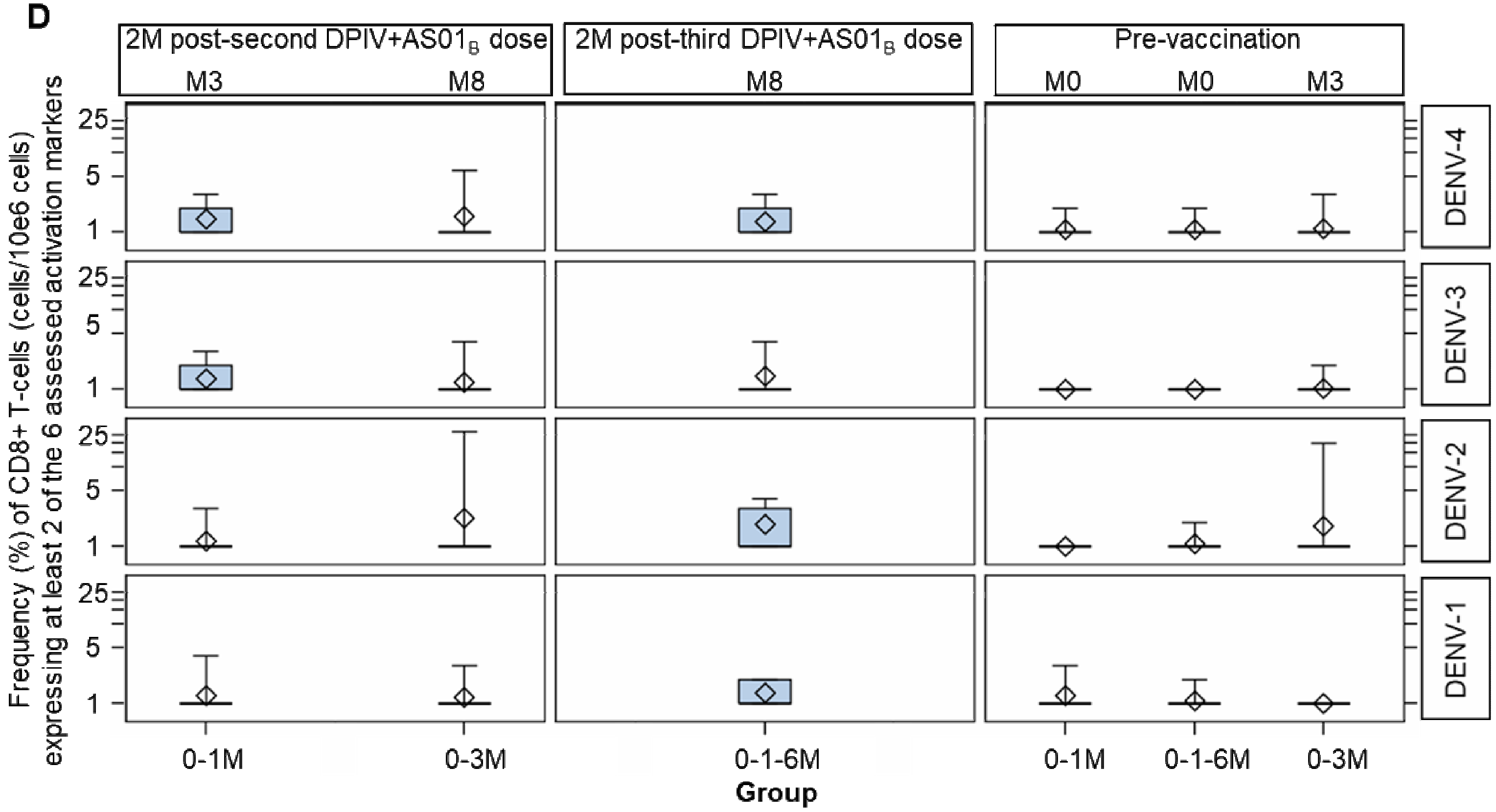

ATP, according to protocol; M, month;DPIV+AS03$_B$, adjuvant system 03$_B$-adjuvanted inactivated tetravalent dengue virus vaccine;DENV, dengue virus; 0–1 M, participants receiving 2 doses of DPIV+AS03$_B$ administered one month apart, at M0 and M1; 0–1–6 M, participants receiving 3 doses of DPIV+AS03$_B$ with first 2 given one month apart and the third given 6 months after the first, at M0, M1 and M6; 0–3 M, participants receiving 2 doses of DPIV+AS03$_B$ administered 3 months apart, at M3 and M6; IL-2, interleukin 2; TNF-α, tumor necrosis factor-alpha.
