# Supplementary figures and images for: Safety and Immunogenicity of an AS03B-Adjuvanted Inactivated Tetravalent Dengue Virus Vaccine Administered on Varying Schedules to Healthy U.S. Adults: A Phase 1/2 Randomized Study
Source: Am J Trop Med Hyg. 2020 Apr 27;103(1):132–41. doi: 10.4269/ajtmh.19-0738 (PMC7356407; doi:10.4269/ajtmh.19-0738)

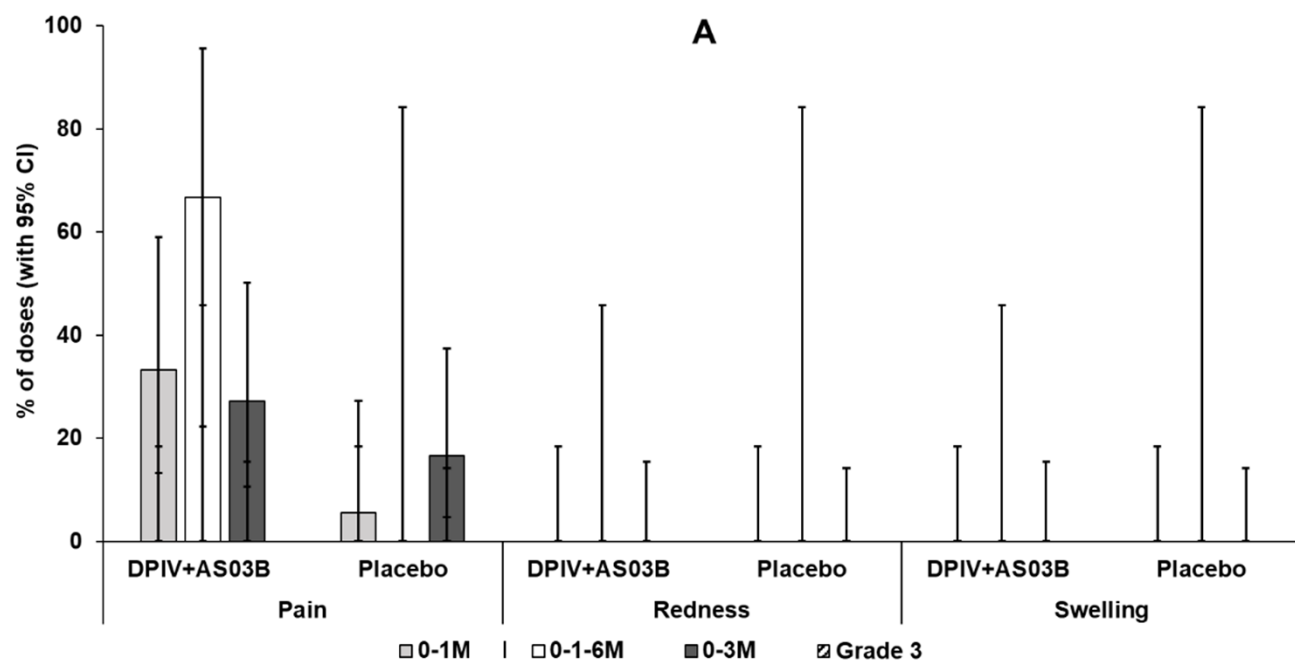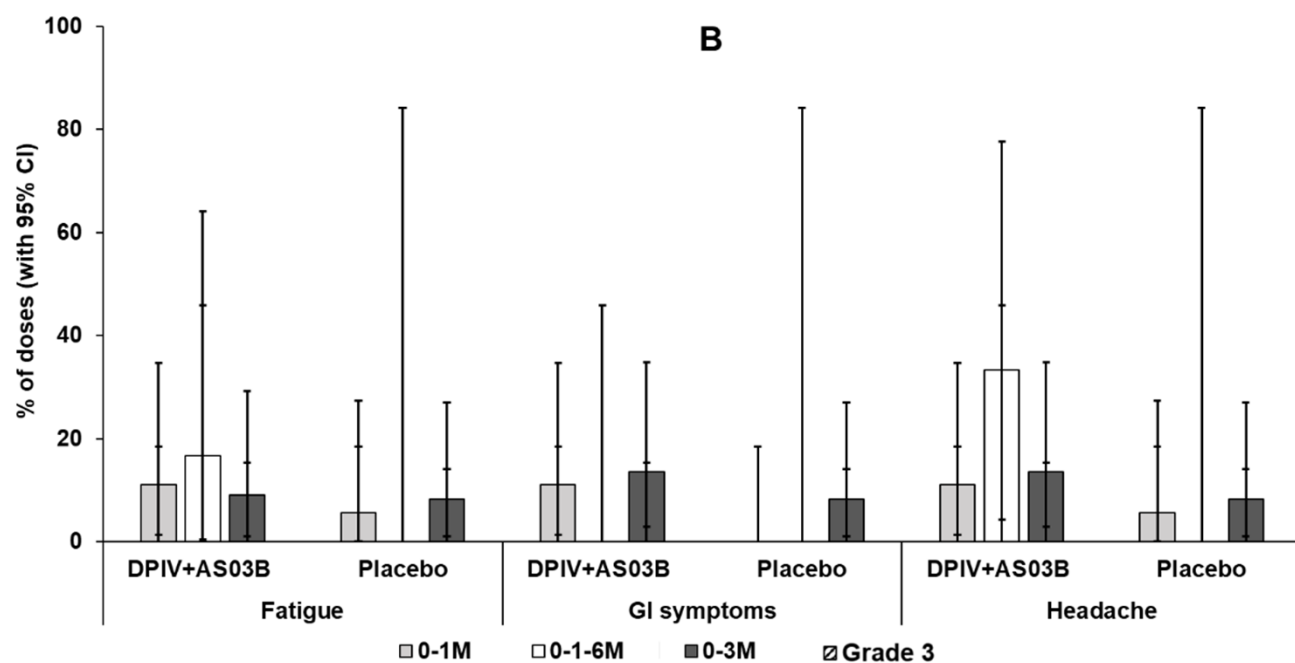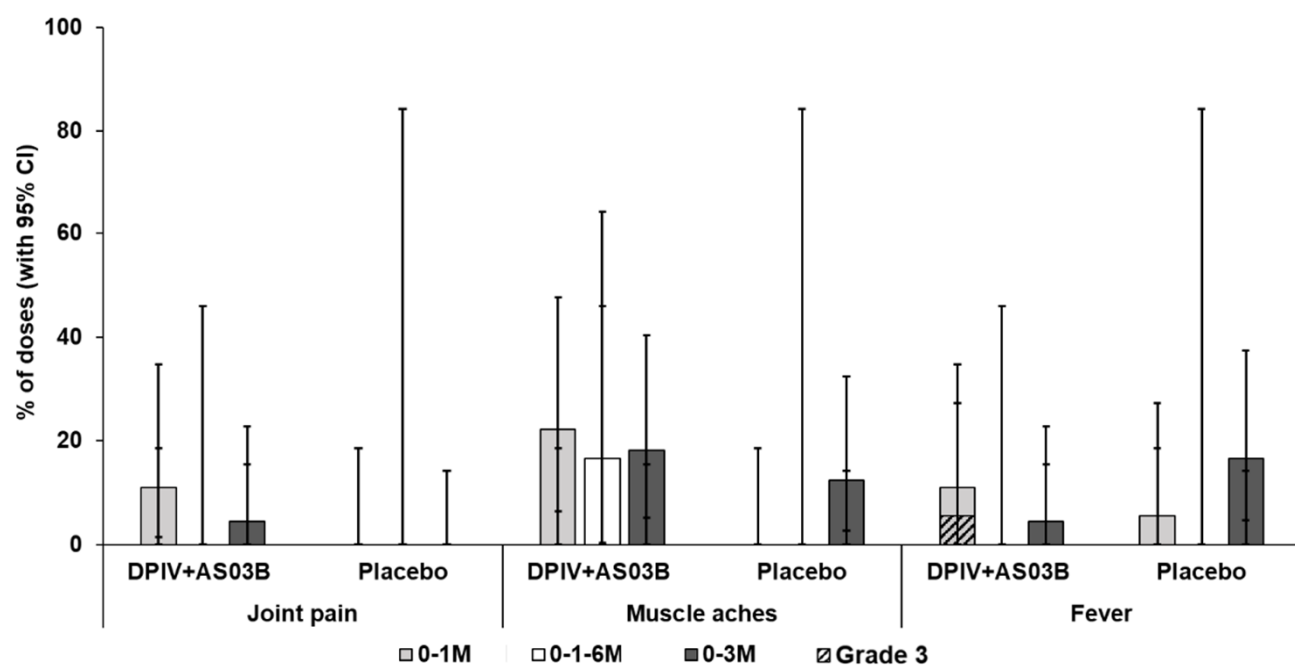

Supplement: Supplementary file 2 [file tpmd190738.SD2.pdf]
