# Supplementary material for: Safety and Immunogenicity of an AS03B-Adjuvanted Inactivated Tetravalent Dengue Virus Vaccine Administered on Varying Schedules to Healthy U.S. Adults: A Phase 1/2 Randomized Study
Source: Am J Trop Med Hyg. 2020 Apr 27;103(1):132–41. doi: 10.4269/ajtmh.19-0738 (PMC7356407; doi:10.4269/ajtmh.19-0738)
Supplement: Supplementary file 3 [file tpmd190738.SD3.pdf]

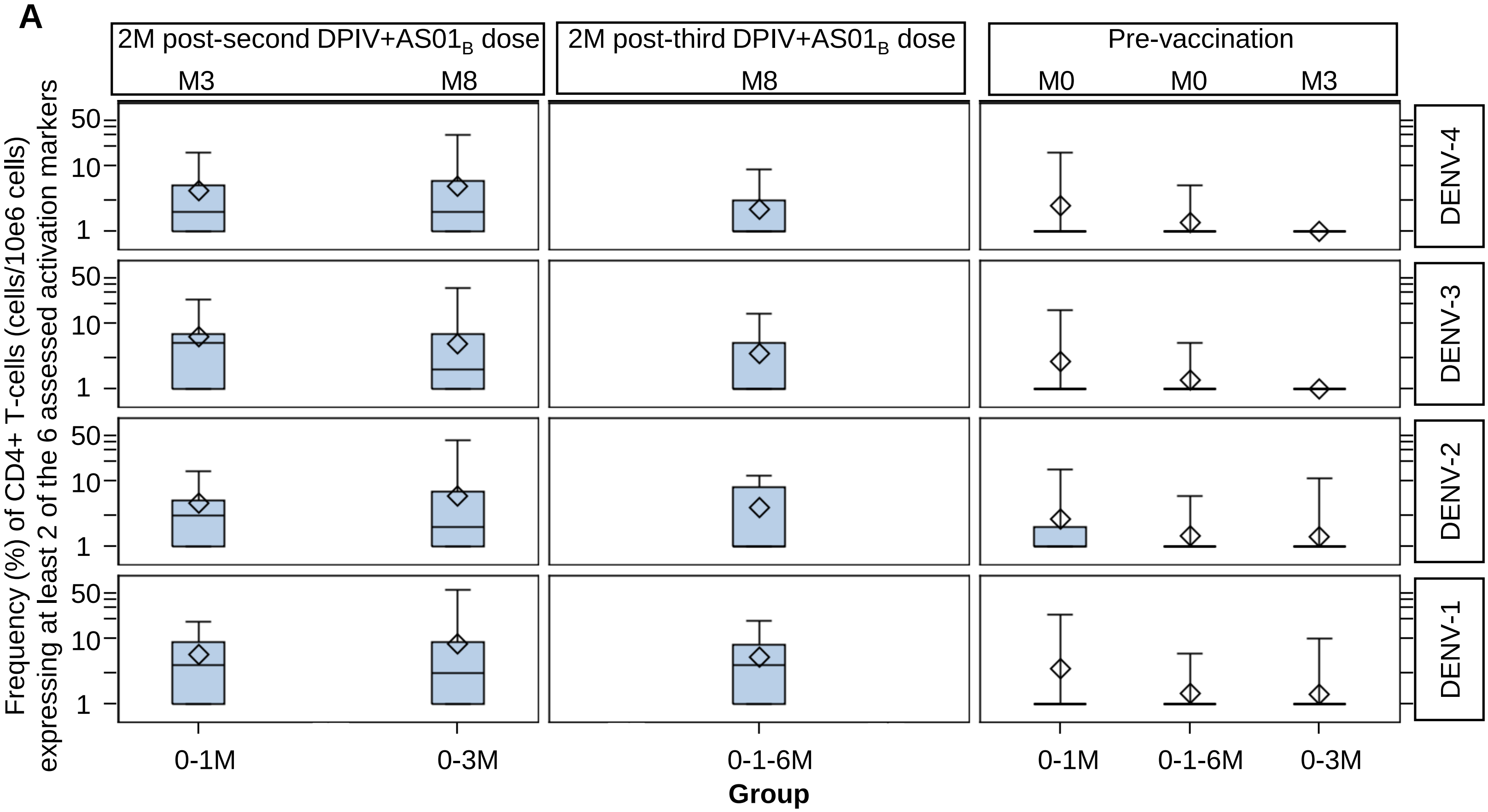
A
2M post-second DPIV+AS01B dose
M3
M8
2M post-third DPIV+AS01B dose
M8
Pre-vaccination
M0
M0
M3
DENV-4
DENV-3
DENV-2
DENV-1
50
10
1
Frequency (%) of CD4+ T-cells (cells/10e6 cells) expressing at least 2 of the 6 assessed activation markers
0-1M
0-3M
0-1-6M
0-1M
0-1-6M
0-3M
Group

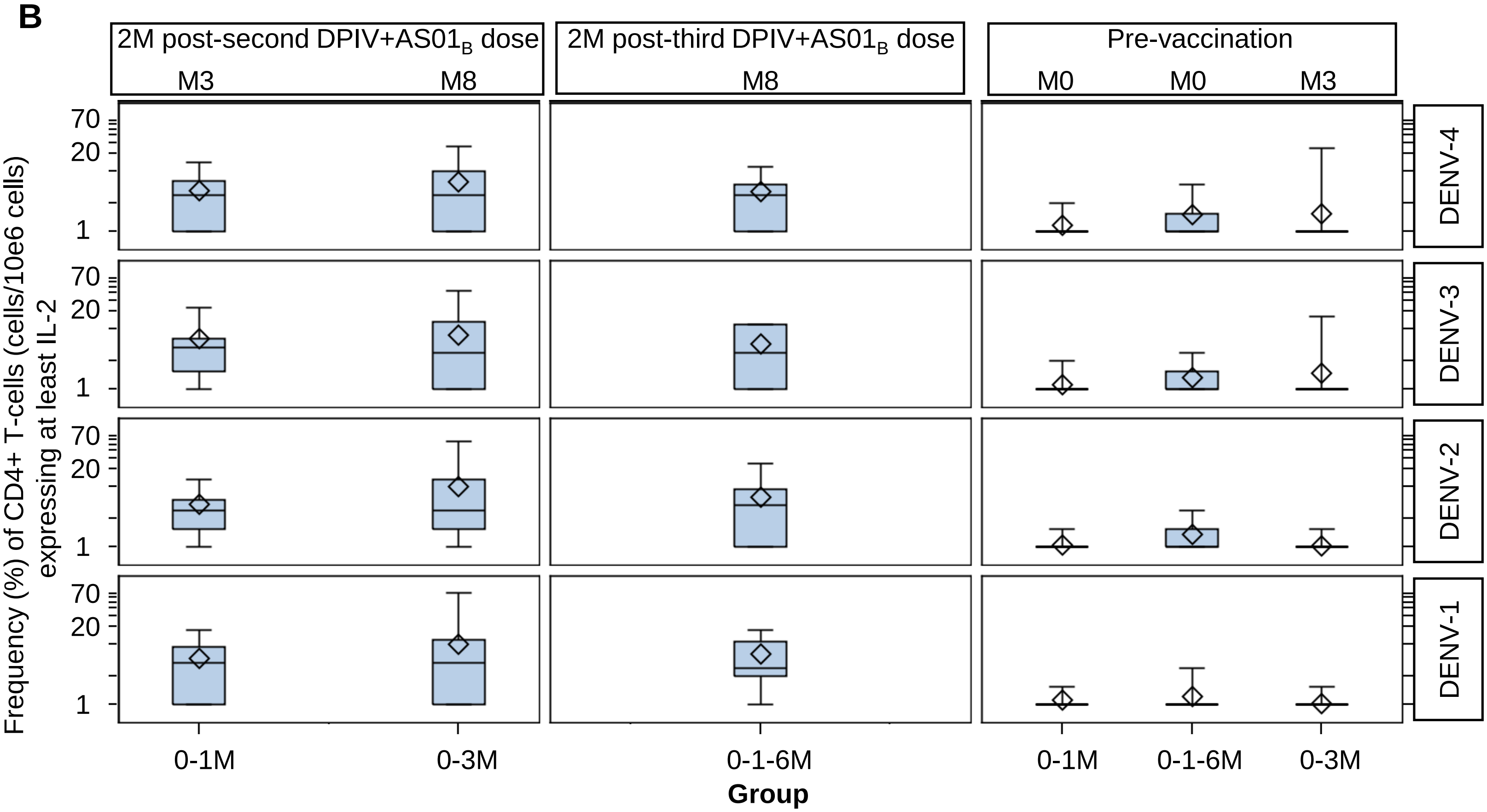

B
2M post-second DPIV+AS01$_B$ dose
M3
M8
2M post-third DPIV+AS01$_B$ dose
M8
Pre-vaccination
M0
M0
M3
DENV-4
DENV-3
DENV-2
DENV-1
70
20
1
Frequency (%) of CD4+ T-cells (cells/10e6 cells) expressing at least IL-2
0-1M
0-3M
0-1-6M
0-1M
0-1-6M
0-3M
Group

C

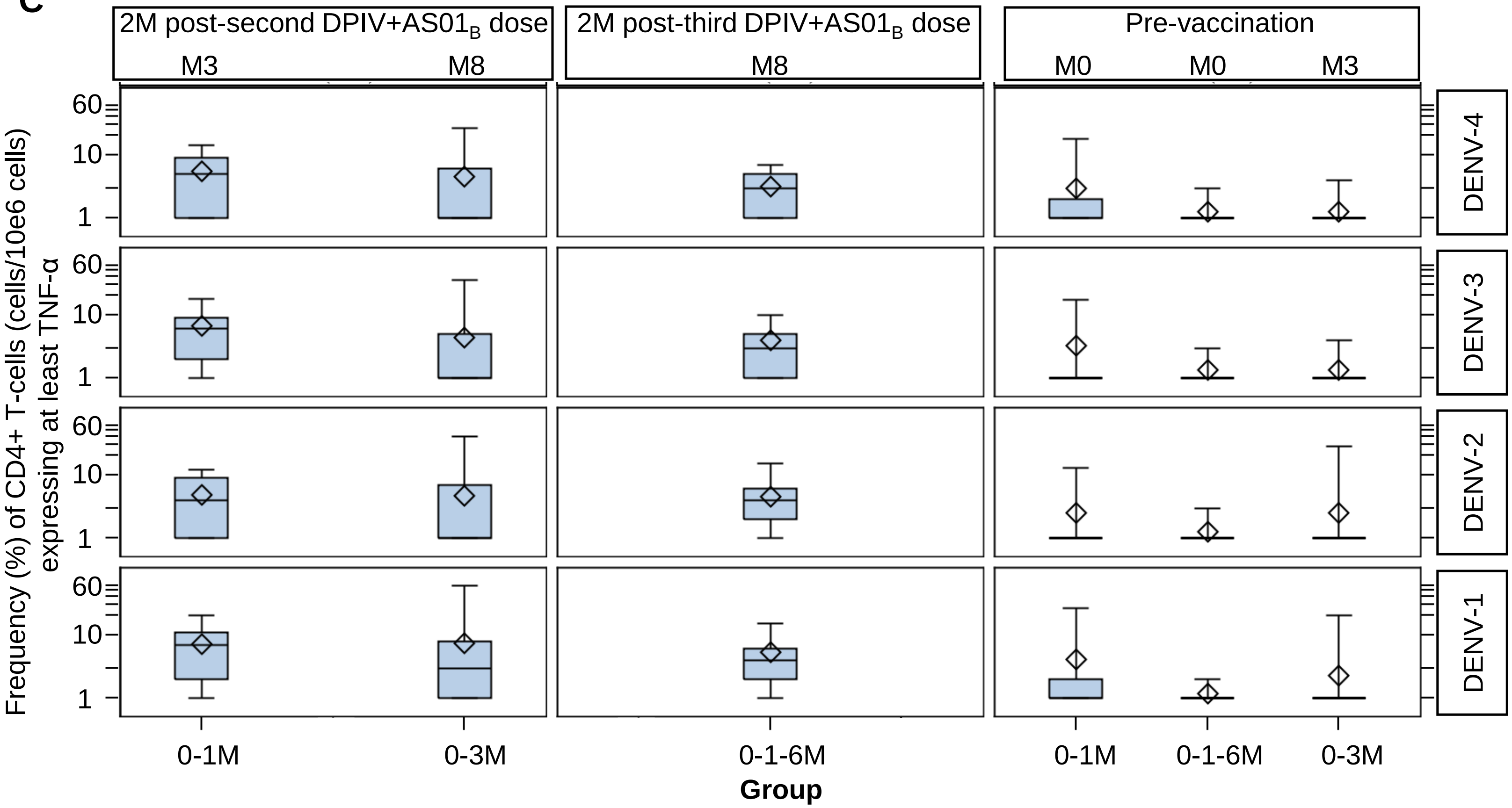

2M post-second DPIV+AS01B dose
M3
M8
2M post-third DPIV+AS01B dose
M8
Pre-vaccination
M0
M0
M3
60
10
1
DENV-4
DENV-3
DENV-2
DENV-1
Frequency (%) of CD4+ T-cells (cells/10e6 cells) expressing at least TNF-α
0-1M
0-3M
0-1-6M
0-1M
0-1-6M
0-3M
Group

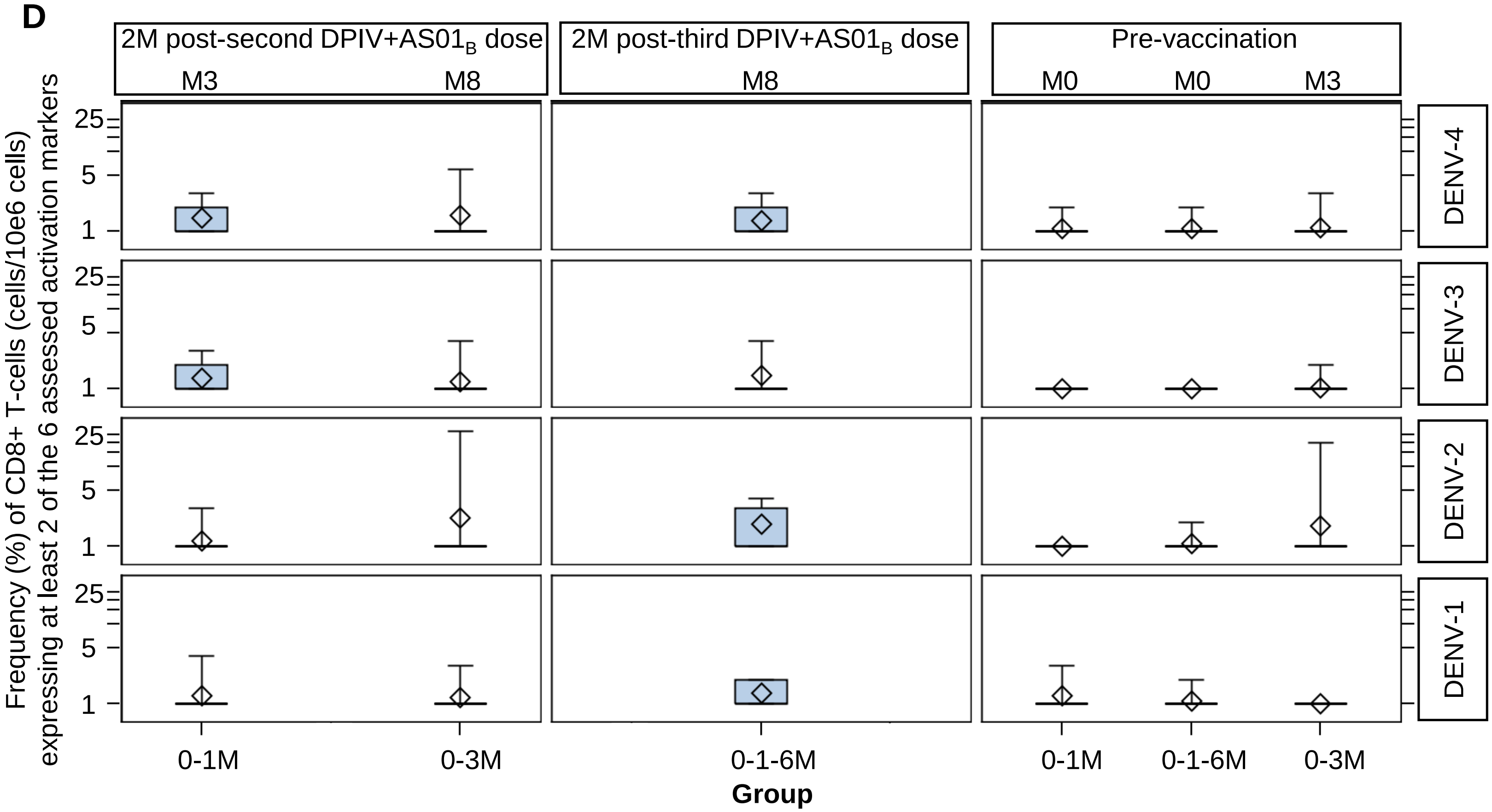
D
2M post-second DPIV+AS01$_B$ dose
2M post-third DPIV+AS01$_B$ dose
Pre-vaccination
M3
M8
M8
M0
M0
M3
DENV-4
DENV-3
DENV-2
DENV-1
25
5
1
Frequency (%) of CD8+ T-cells (cells/10e6 cells) expressing at least 2 of the 6 assessed activation markers
0-1M
0-3M
0-1-6M
0-1M
0-1-6M
0-3M
Group
